# Supplementary material for: Unexpected PD‐L1 immune evasion mechanism in TNBC, ovarian, and other solid tumors by DR5 agonist antibodies
Source: EMBO Mol Med. 2021 Feb 15;13(3):e12716. doi: 10.15252/emmm.202012716 (PMC7933954; doi:10.15252/emmm.202012716)
Supplement: Supplementary file 1 — Appendix [file EMMM-13-e12716-s001.docx]

**Appendix File**

Appendix File Table of contents:

1) Appendix Figure S1 to S11

2) Appendix Figure Legends

3) Appendix Table S1 to S4

4) References for material and methods

**1) Appendix Figures S1-S11**

**
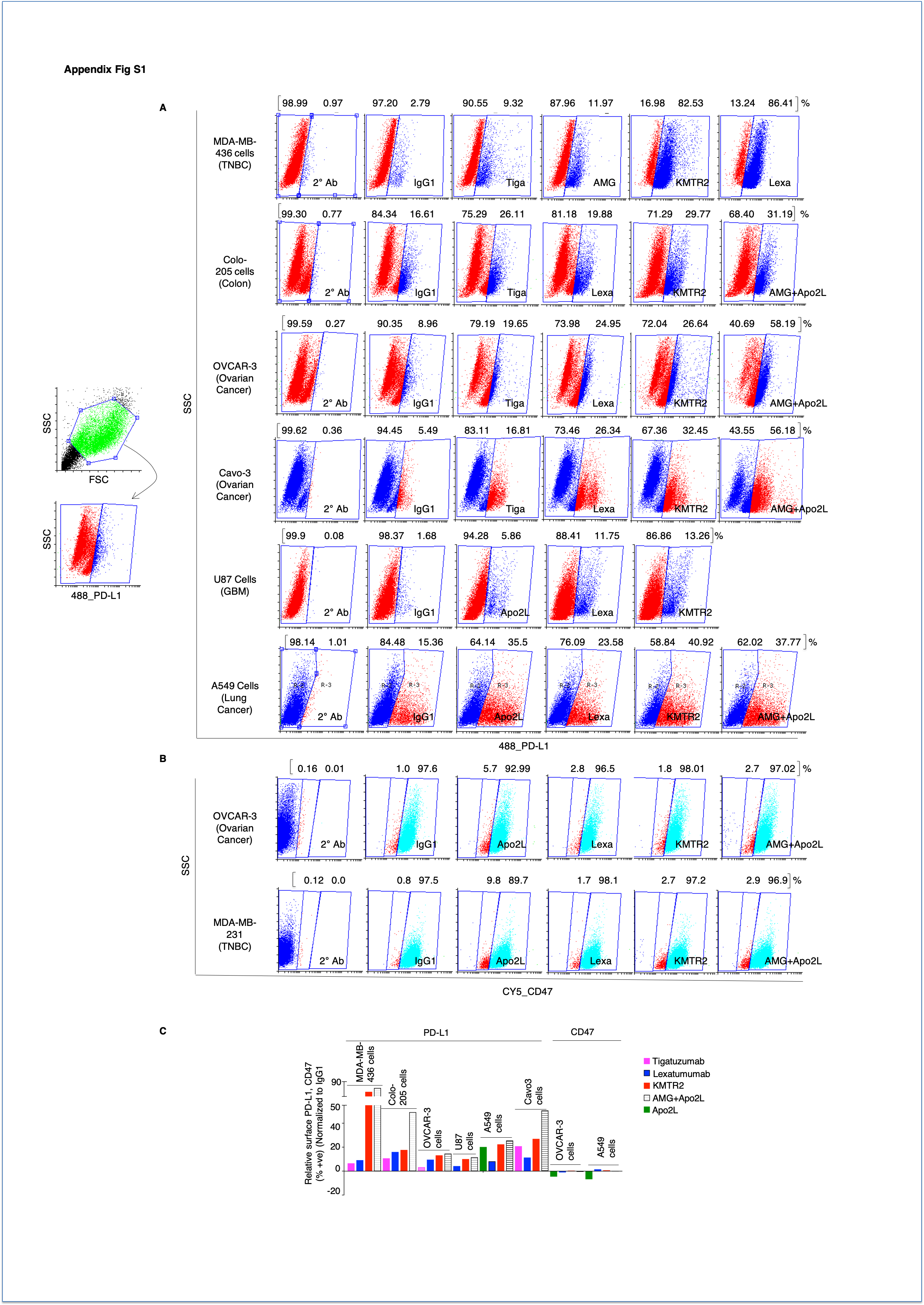
**

**
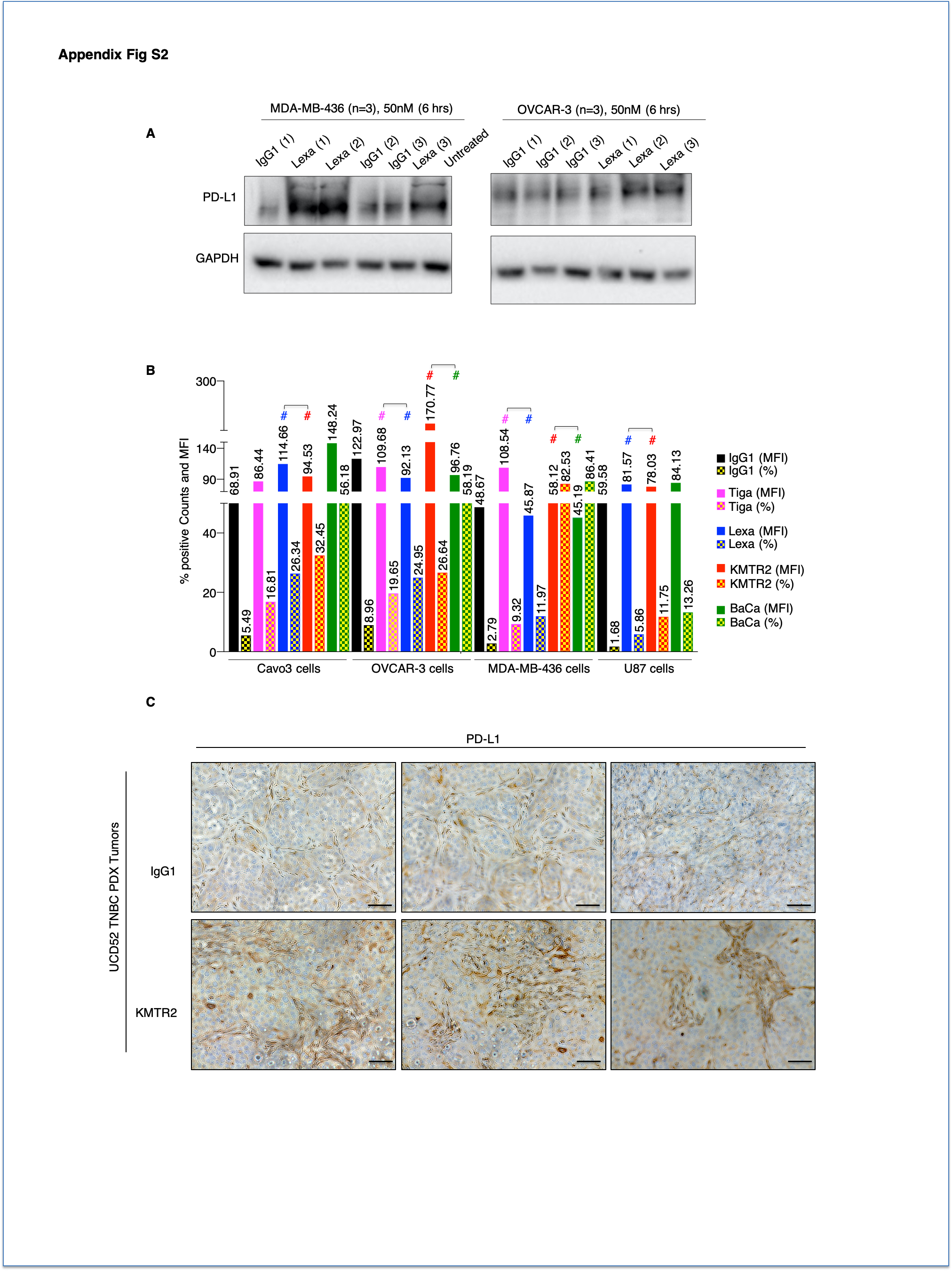
**

**
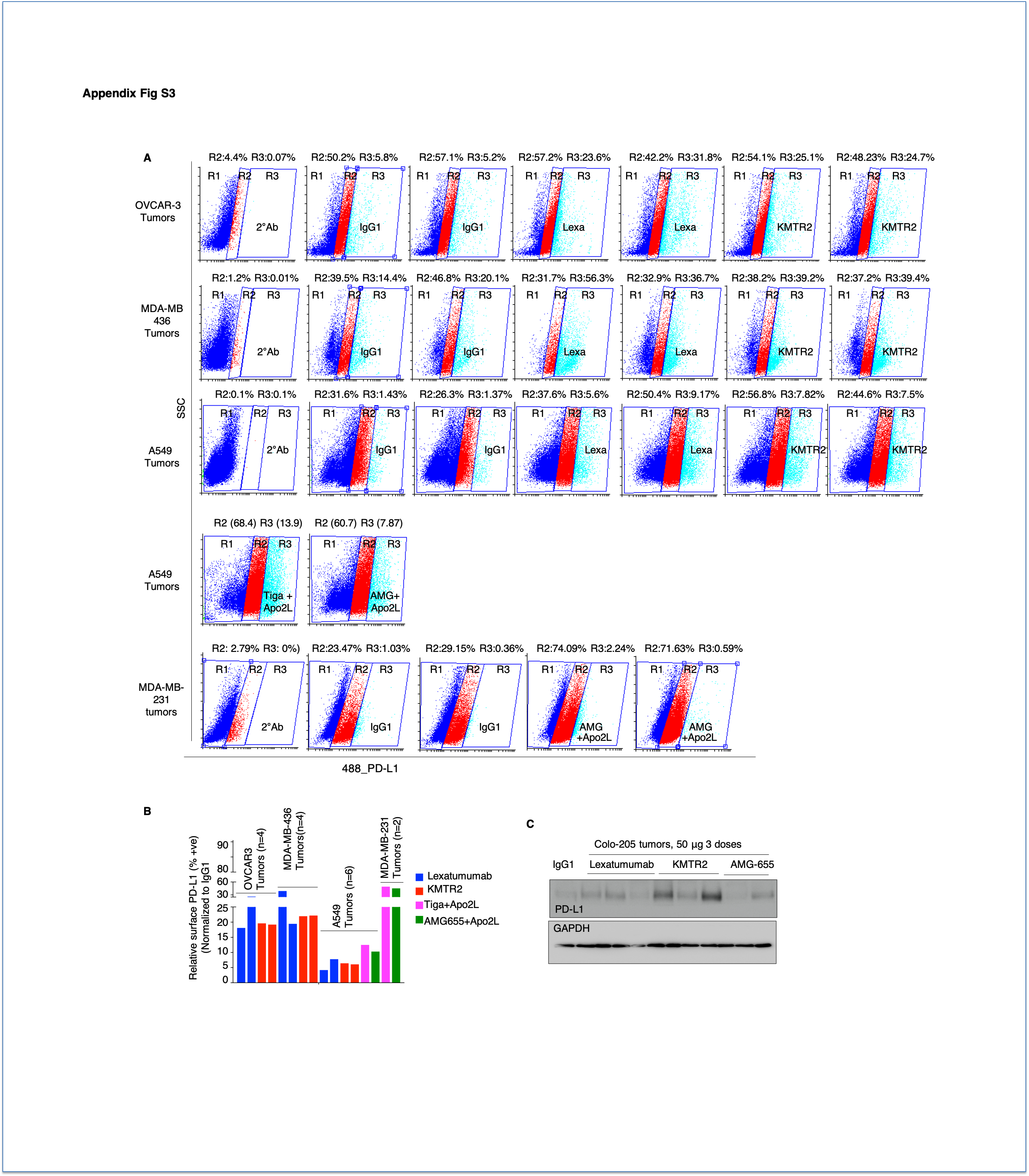
**

**
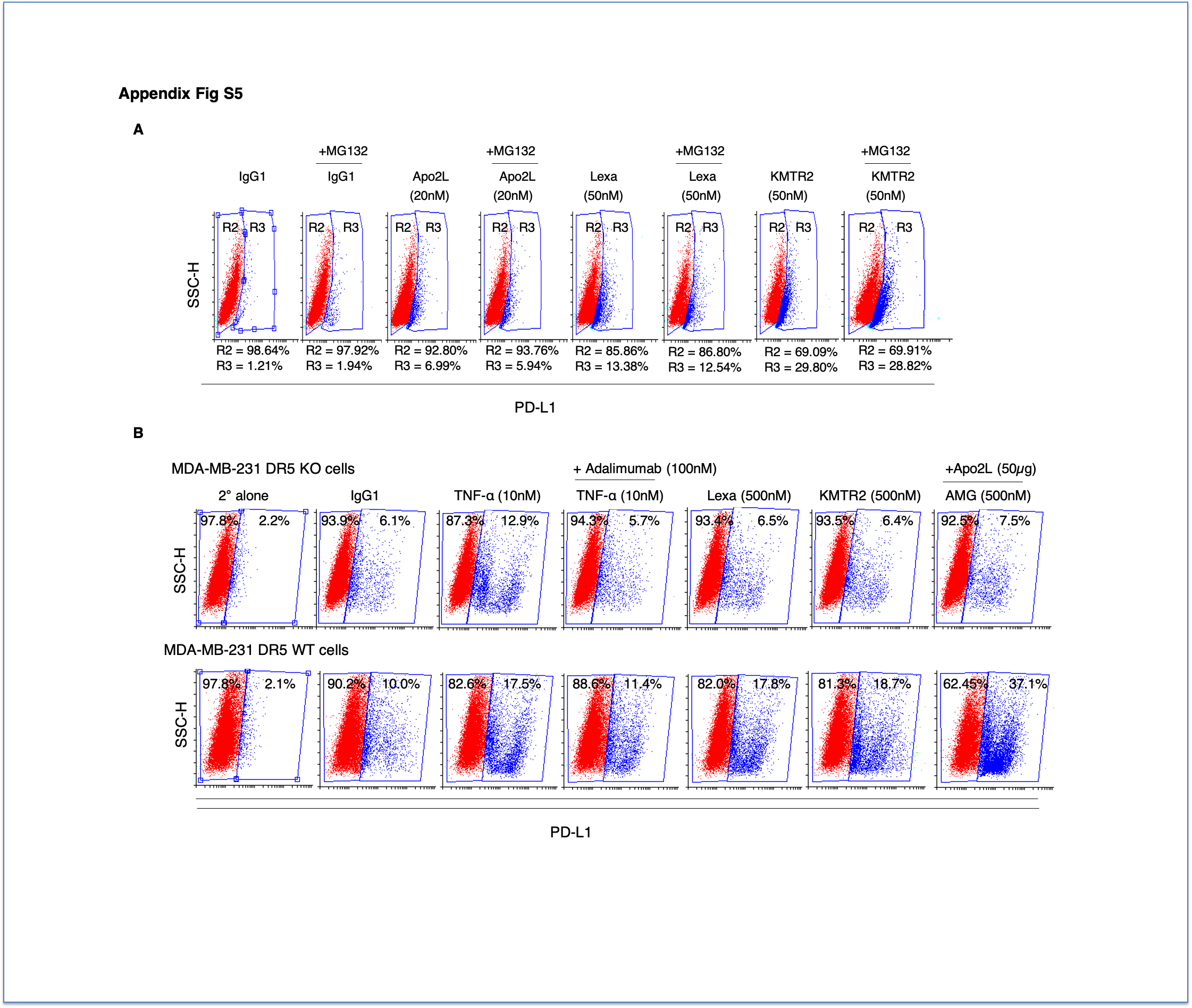
**

**
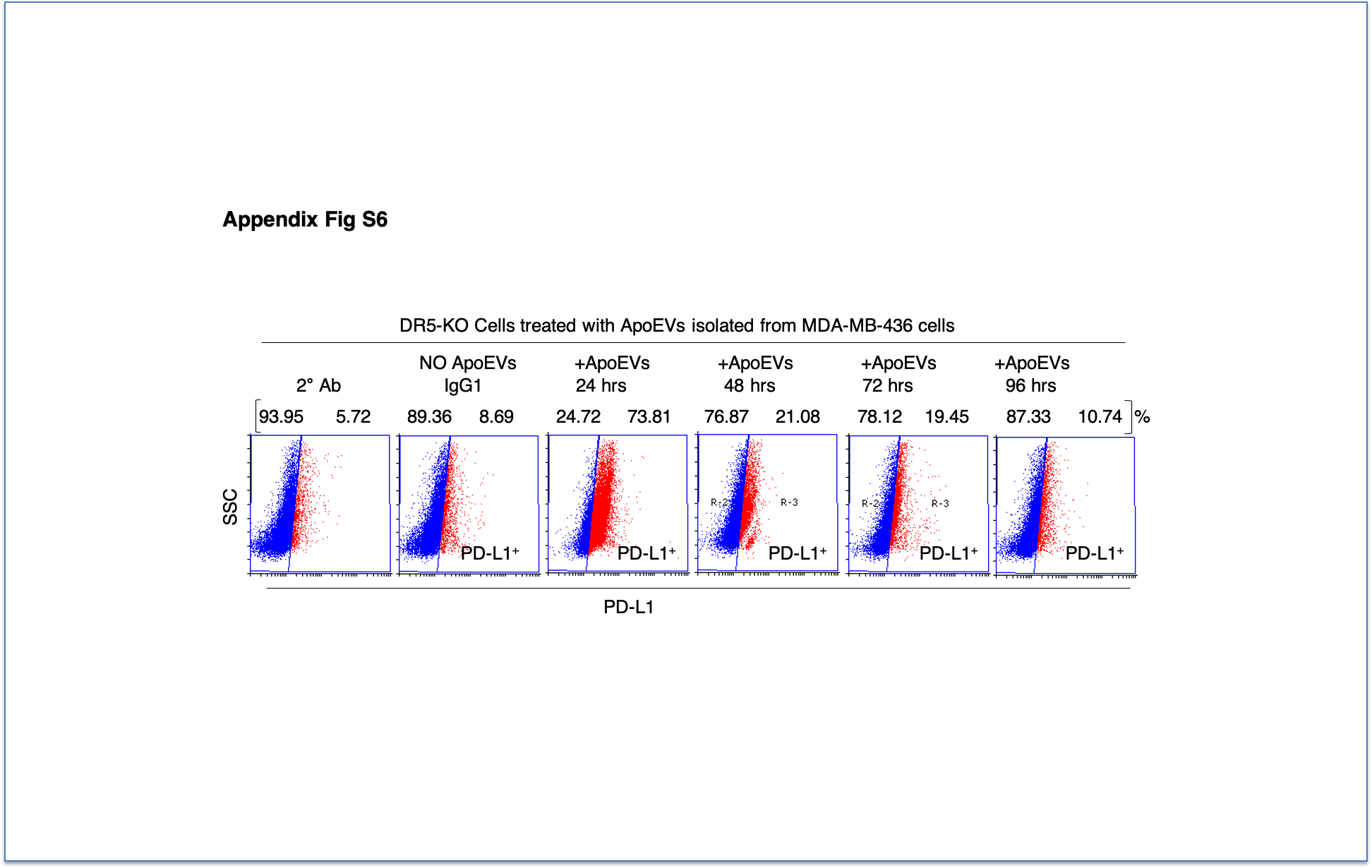
**

**
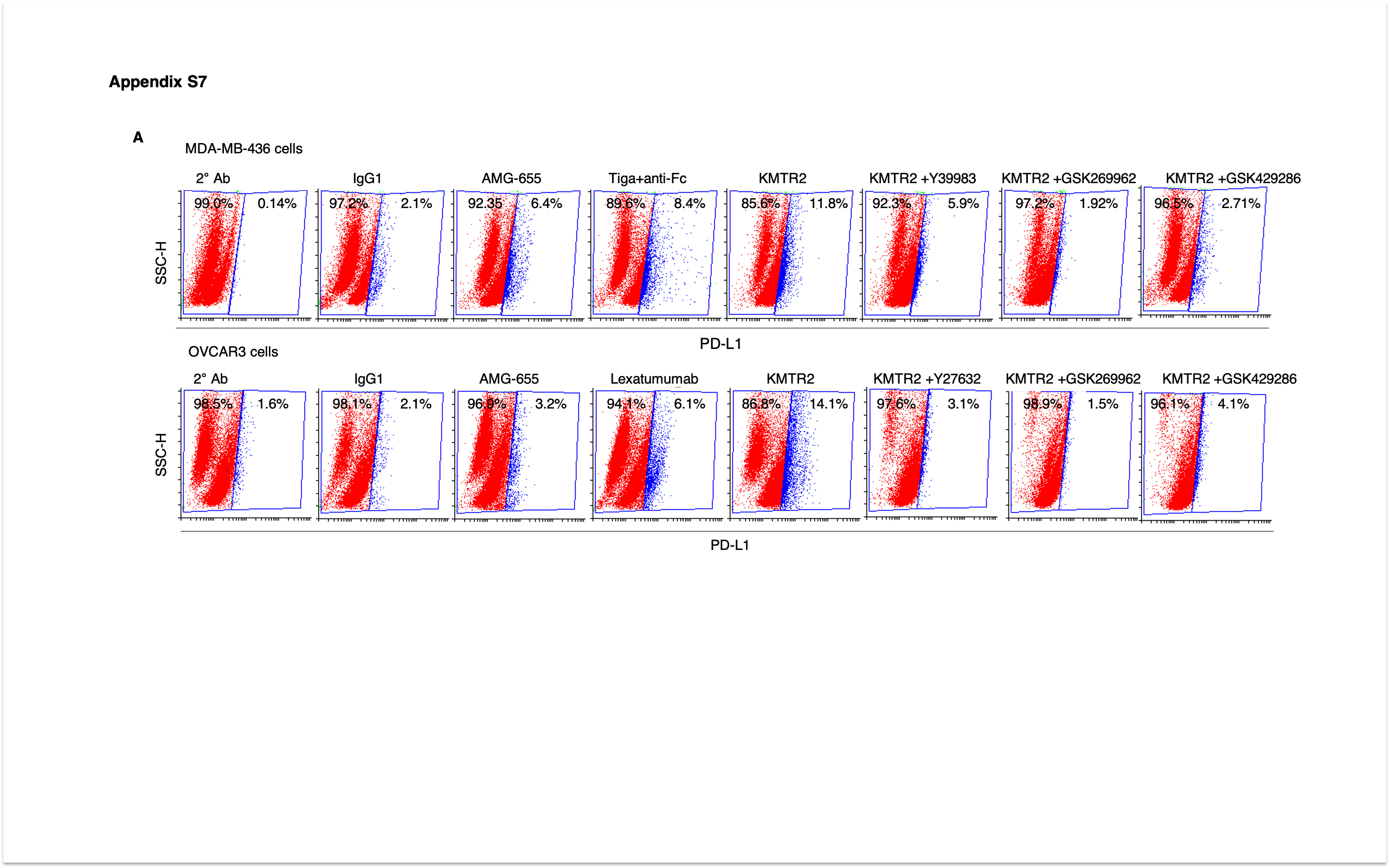
**

**
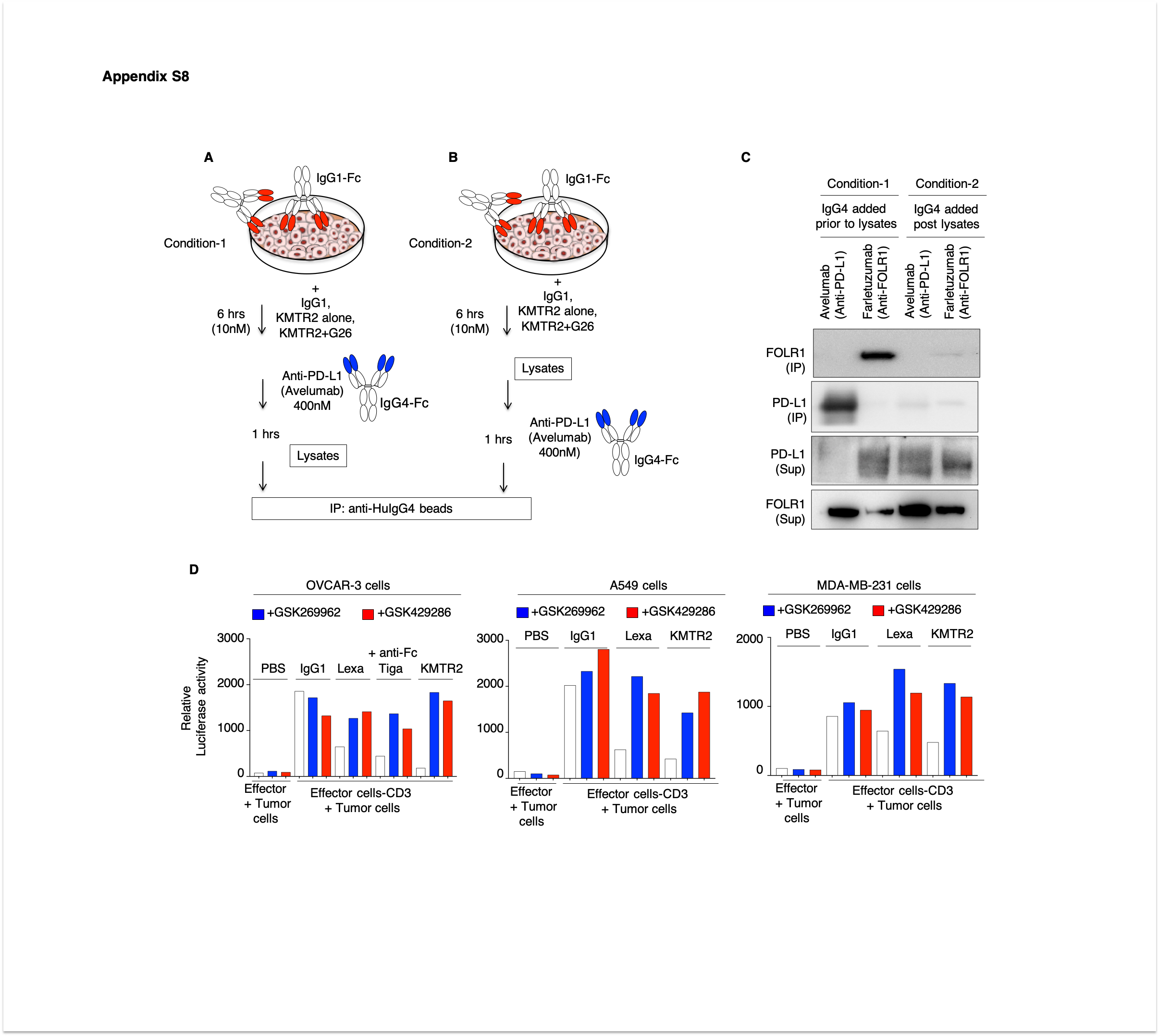
**

**
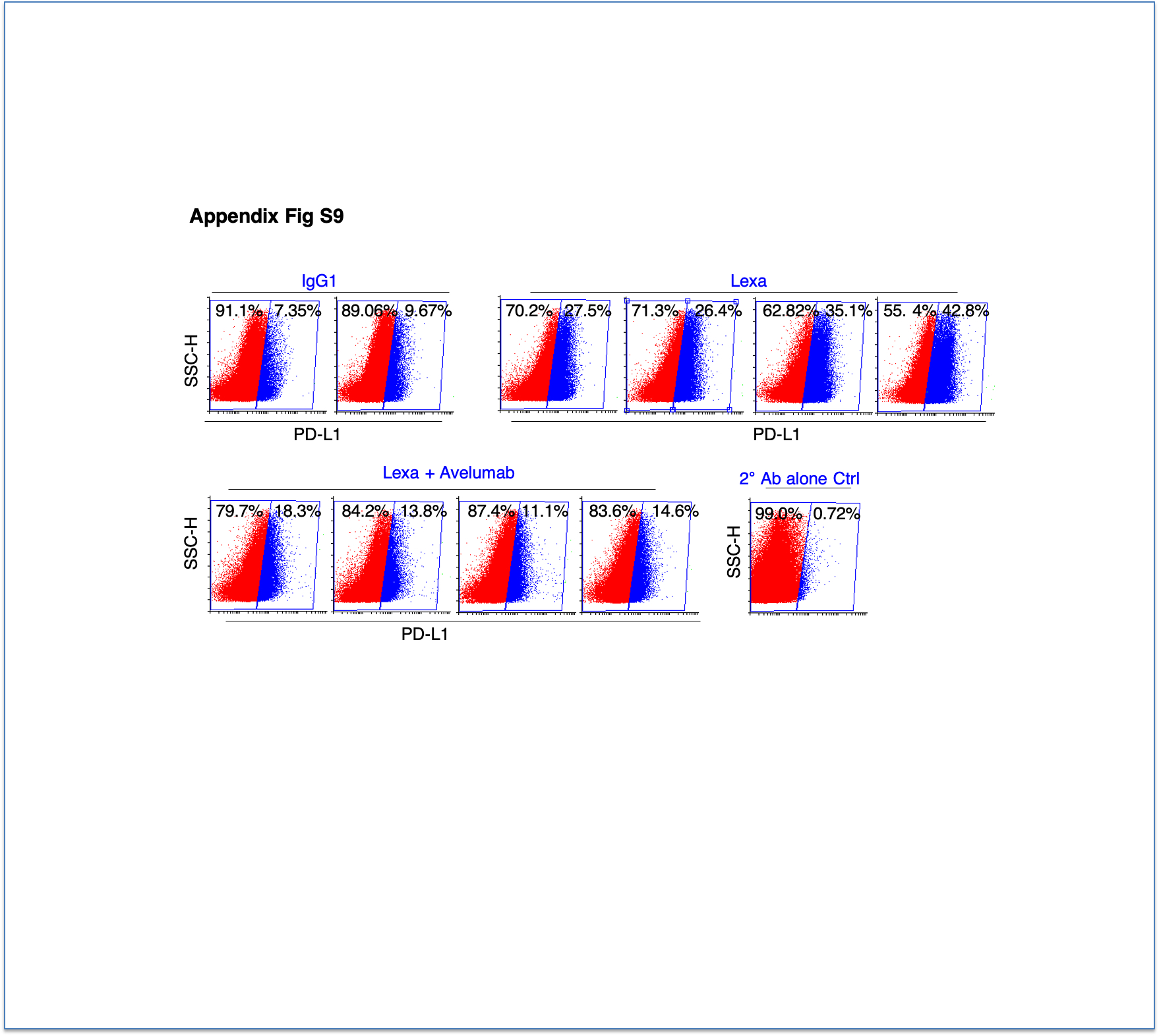
**

**
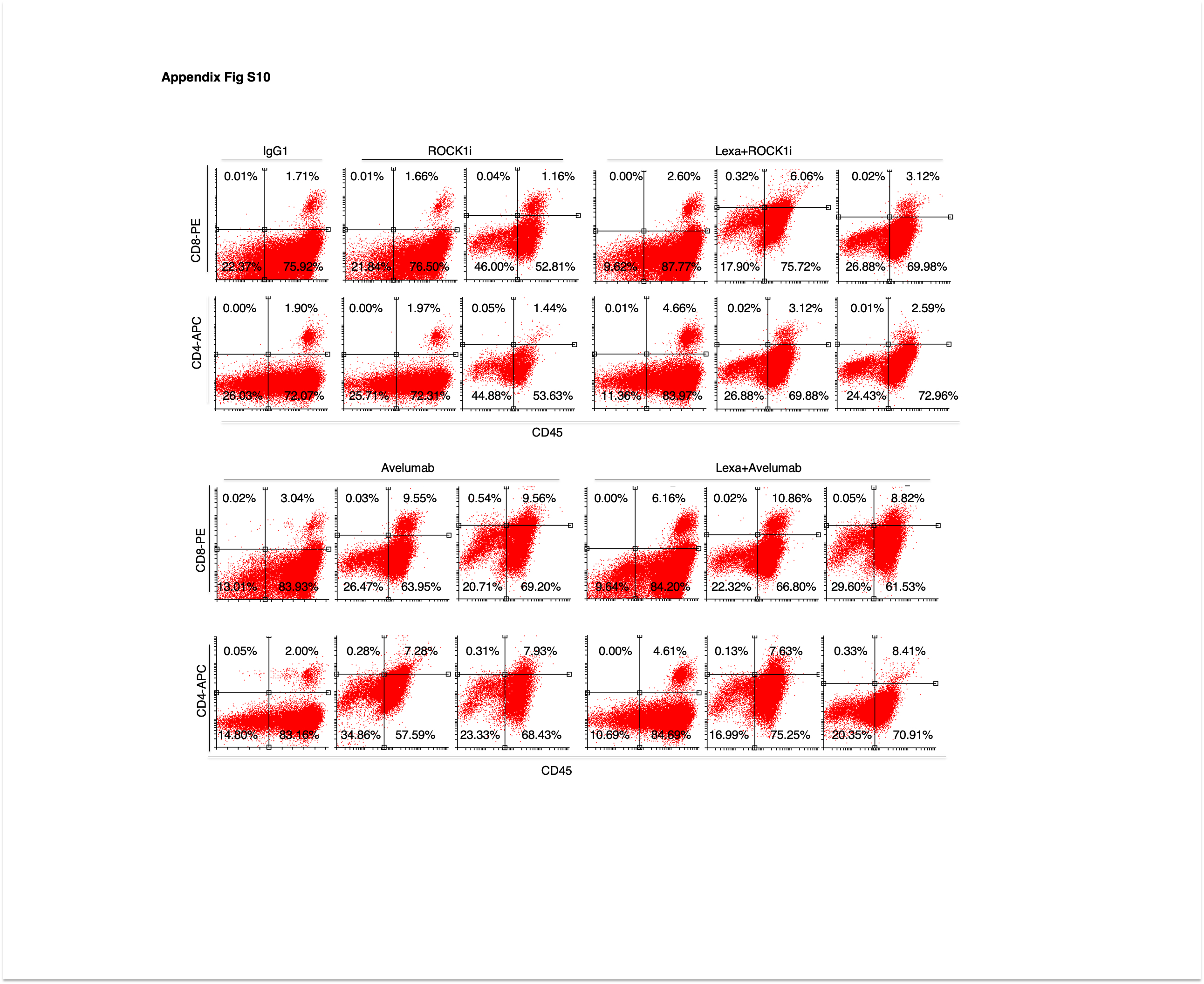
**

**
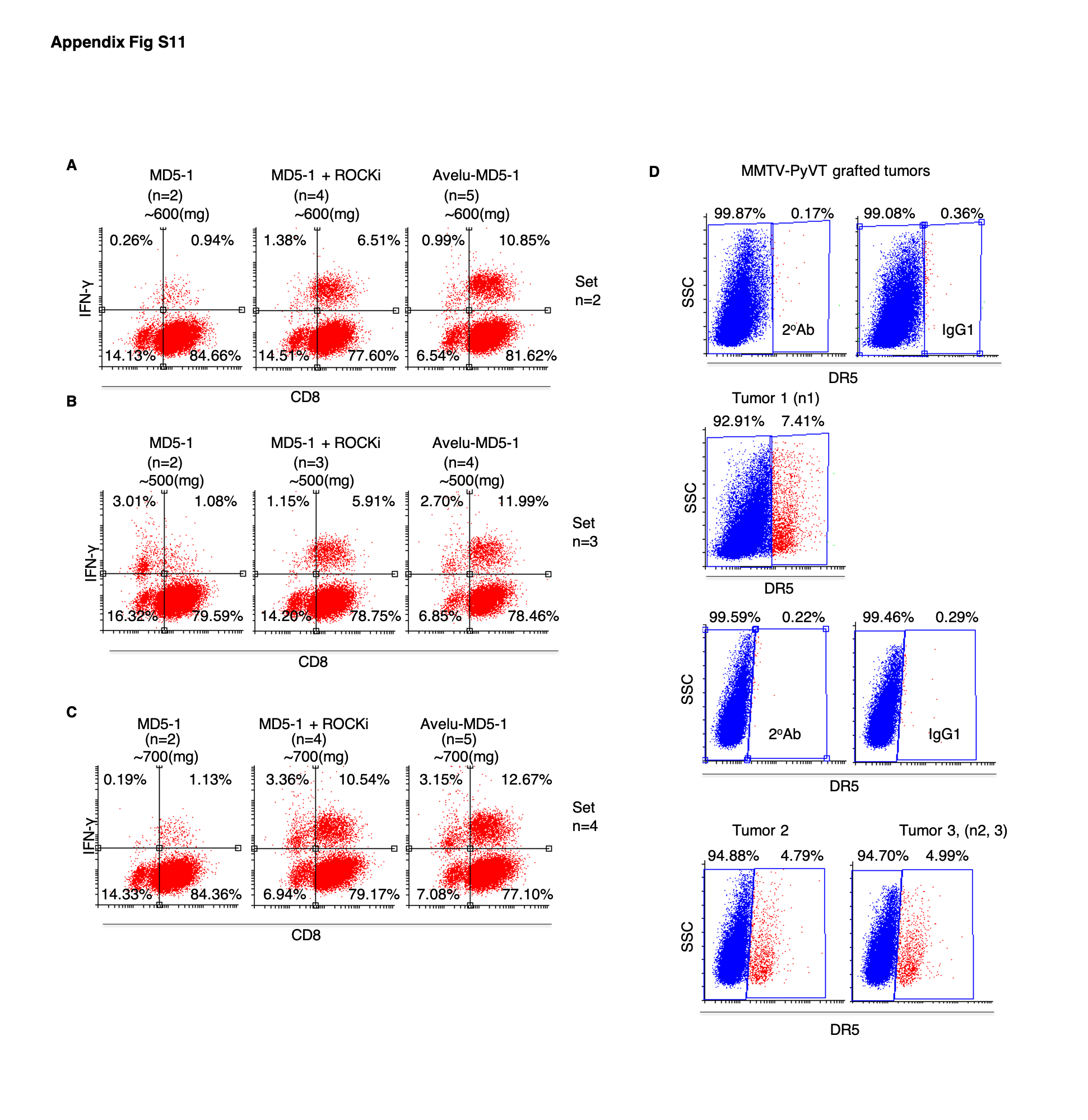
**

**2) Appendix Figure Legends**

**Appendix Fig S1**

(A-B) Various solid tumor cells lines (MDA-MB-436, MDA-MB-231, OVCAR3, Cavo-3, U87, A549 etc.) treated with indicated DR5 agonist (± anti-Fc) for 4-8 hours were analyzed for surface PD-L1 using flow cytometry. (B) MDA-MB-231, OVCAR3 treated with DR5 agonist for 4-6 hours were analyzed for surface CD47.

(C) Flow cytometry analysis showing relative PD-L1 and CD47 surface intensity from indicated tumor cell lines treated with indicated DR5 agonists (normalized to IgG1 treatment). Since each cell lines have been treated with at least 3 DR5 agonist antibodies (lexatumumab, tigatuzumab, KMTR2, AMG655+Apo2L), normalized surface PD-L1 and CD47 represent biological replicate (n=3).

**Appendix Fig S2**

(A) 3 biological replicates of total PD-L1 and GAPDH from triple negative breast cancer cell (MDA-MB-436) and ovarian cell (OVCAR-3) lysates treated with lexatumumab for 6 hrs.

(B) Raw mean fluorescent intensity (MFI) and % positive PD-L1 counts from the cells treated with indicated DR5 agonist antibodies. MFI values are shown on the top of solid color bars. There are multiple instances (shown on the top) where despite having lower % positive PD-L1 population, MFI values were higher after indicated DR5 agonist treatments. As an example, in MDA-MB-436 cells treated with tigatuzumab and lexatumumab, lexatumumab treated cells had higher % positive PD-L1 population (9.32 vs 11.97), however MFI was higher in tigatuzumab treated population (107.54 vs 45.87).

(C) KMTR2 treated (50μg, 4 doses) UCD52 TNBC PDX tumors were stained for PD-L1 using immunohistochemistry (IHC). 3 additional representative images are shown. Also see Fig 1L and material and methods. Representative images from n=2 tumors.

**Appendix Fig S3**

(A) 0.5x10^6^-2x10^6^ indicated tumor cells were injected subcutaneously in NOD.Cg Prkdc^scid^ Il2rg^tm1Wjl^/SzJ animals with Matri in PBS. When tumors appeared on animals (3-4 weeks), animals were i.p. injected with indicated DR5 agonists (4-6 doses only), followed by tumor excision and single cell suspension isolation from tumors after indicated antibody treatments. Isolated tumor cells were analyzed for surface PD-L1 using flow cytometry.

(B) Raw Data shown in (A) was normalized with % positive PD-L1 in IgG1 treated tumors (n=2-5 tumors)

(C) Colo-205 tumor harboring athymic Nude *Foxn1^nu^/Foxn1^+^* (Envigo) xenografts were treated with IgG1, lexatumumab, KMTR2 and AMG655 (3 doses, 100μg each). Harvested tumor lysates were analyzed for total PD-L1 expression. GAPDH is leading control.

**Appendix Fig S4**

(A) Effector jurkat cells stably expressing human PD-1 and luciferase under the response element (RE) of nuclear factor of activated T-cells (NFAT) are shown in green, while antigen presenting cells CHO-K1 (APC/CHO-K1) stably expressing human PD-L1 are shown in blue. In co-cultures, PD-L1-PD-1 interaction results in downregulation of NFAT-RE signaling. As a result, luciferase activation is blocked hence low or no substrate signal (Glo). Blockade of PD-L1-PD-1 interaction by corresponding antibodies eliminates downregulation of NFAT-RE signaling. This results in increased luciferase activity.

(B) Modified PD-1-PD-L1 interaction model for our studies: (1) Engagement T-cell receptor’s (TCR) CD3 subunit (by anti-CD3 antibody) activates luciferase in PD-1 effector jurkat reporter cells. (2) Upon DR5 agonist treatment, tumor cells mobilize PD-L1 on cell surface. (3) When DR5 agonist treated tumor cells are co-cultured with CD3 activated Jurkat cell, surface mobilized PD-L1 engages PD-1 on jurkat reporter cells leading to loss of luciferase activity.

(C) Tumor cell-Jurkat cell co-culture assay in Colo-205, MDA-MB-231, U87 and A549 cells after treatment with indicated DR5 agonists. Because of their higher sensitivity to cell-death, for Colo-205 and U87 cells 100nM conc. of DR5 agonist was used. Because of their lower level of sensitivity to cell-death, for MDA-MB-231 and A549 cells 500nM conc. of DR5 agonist was used.

(D) OVCAR3 cells treated with KMTR2 for indicated times were lysed and immunoblotted as indicated for PD-L1, PARP, CMTM6, Total p65, Total STAT3, Total ERK and E-cadherin.

**Appendix Fig S5**

(A) PD-L1 flow cytometry analysis of MDA-MB-436 cells were treated with IgG1 control ±MG132, Apo2L ±MG132, lexa ±MG132 and KMTR2 ±MG132 with indicated concentrations. After indicated treatments (on the top), cells were analyzed for surface PD-L1. R2 indicates % MDA-MB-436 cells positive with basal surface PD-L1 levels (shown at bottom) and R3 indicates % of cells positive for surface PD-L1 after indicated treatments (shown at bottom).

(B) WT MDA-MB-231 cells or MDA-MB-231 DR5-KO cells treated with DR5 agonist (as indicated on top) and TNF-α next to each other followed by PD-L1 analysis using flow cytometry.

**Appendix Fig S6**

ApoEVs were isolated from DR5-WT MDA-MB-436 cells after 8 hours of KMTR2 treatment as described in material and methods. Isolated ApoEVs were added on to DR5-KO (MDA-MB-231) cells for indicated times (24-96 hrs) to analyze ApoEVs mediated PD-L1 transfer kinetics in DR5-KO cells. Secondary antibody (2°) alone and no ApoEVs are controls. Highest surface PD-L1 signal was evident on DR5-KO (MDA-MB-231) cells after 24hrs (73.81%) of ApoEVs treatment. Top of each FACs dot plot the % cells positive for PD-L1 (Red dots) are shown along with % cells expressing basal PD-L1 (Blue dots)/

**Appendix Fig S7**

MDA-MB-436 (Top) and OVCAR3 (Bottom) cells were pre-treated with indicated ROCK1 inhibitors. After 2 hours of ROCK1 inhibitor treatment, cells were treated indicated DR5 agonists antibodies (20nM) for 4 more hours. Cells were later analyzed for surface PD-L1 using flow cytometry. Tiga (tigatuzumab), AMG-655, Lexatumumab and KMTR2 are DR5 agonists. Y39983, GSK269962, GSK429286 are ROCK1 inhibitors.

**Appendix Fig S8**

(A-B) Illustration of immunoprecipitation using clinical anti-PD-L1 avelumab antibody as described in Fig. 4F-H. Condition 1: IgG1 Fc containing DR5 agonists (KMTR2 or other controls) were added on tumor cells either alone or after pre-treatment of ROCK1 inhibitor (GSK269962A) for 6 hrs. This was followed by lysates preparation in low salt RIPA buffer. Lysates were later incubated with IgG4 Fc containing avelumab for 1 hr, followed by pull down of avelumab using IgG4 Fc specific beads. Condition 2: IgG1 Fc containing DR5 agonists (KMTR2 or other controls) were added on tumor cells either alone or after pre-treatment of ROCK1 inhibitor (GSK269962A) for 6 hrs. After 6 hrs, IgG4 Fc containing avelumab was added to the media for additional 1 hr to bind to surface mobilized PD-L1. Lysates were made in low salt RIPA buffer, followed by pull down of avelumab using IgG4 Fc specific beads.

(C) After separating the lysates on SDS-PAGE, immunoblotting was carried out using commercial PD-L1 antibody. In similar condition, another clinical anti-FOLR1, farletuzumab was used, followed by immunoblotting using commercial FOLR1 antibody. Only in condition 1, clinical antibodies pull down bound native protein complexes.

(D) Tumor cell-Jurkat cell co-culture assay using A549, MDA-MB-231 and OVCAR3 tumors cells after treatment with indicated DR5 agonists alone or GSK269962 and GSK429286 (ROCK1 inhibitors) pretreated cells. Increased average luciferase intensity after ROCK1 inhibitor treatment from reporter cells confirmed decreased PD-L1 surface mobilization.

**Appendix Fig S9**

Chi-G4S-DR5 stable 4T1 tumors were treated with indicated DR5 agonist lexatumumab either alone or in combination of avelumab (5 doses total). Recovered tumor cells were analyzed for surface PD-L1 expression using flow cytometry similar to the experiment described in Fig 1I-J

**Appendix Fig S10**

Chi-G4S-DR5 stable 4T1 tumors harboring mice were treated lexatumumab, lexatumumab +ROCK1i, and avelumab + lexatumumab and other controls as indicated. Antibodies were treated i.p at 100μg dose (6 total), ROCK1i (in PBS) was injected directly near tumors at 2mg/kg dose (6 total). Harvested tumors were grouped together and sized matched (3 independent sets: n=2-6 tumors in each set) followed by TIL isolation (see methods). CD8/CD45 and CD4/CD45 expressing cells were measured by flow cytometry. The data shown here is from two additional set of experiment. See also Fig 6A-C

**Appendix Fig S11**

(A-C) 6-8 weeks old C57BL/6 mice bearing MC38 tumors were intraperitoneally (i.p.) injected with 50μg of indicated antibody every third day. On day 18, tumors were harvested, sized matched and pooled by treatment group, exposed to collagenase/DNase and were single cell suspensions enriched for CD8+ cells. Enriched CD8+ T-cells from various treatments were restimulated with anti-CD3 (OKT3) antibody for 4 additional hours. CD8 gated cells were next analyzed for IFN-γ intracellular expression using flow cytometry. The data shown is from three additional set of experiments. See also Fig 6L-M.

(D) Frozen FVB/N-Tg(MMTV-PyVT)634Mul/J tumor cells (isolated from Stock No: 002374 animal tumors, Jackson Laboratory) were provided by Dr. Paula Bos and were grafted on C57BL/6 animals. After reaching ~400mm^3^, grafted GEM tumors were extracted from animals and single cell suspension was isolated as described in Fig 1I. Isolated tumor cells were analyzed for surface DR5 using flow cytometry (n=3)

**3) Appendix Table S1 to S4**

**Appendix Table S1.**

Critical antibodies and other reagents used and their corresponding activating and inhibitory functions

| Reagent name | Type | Function |
| --- | --- | --- |
| GSK269962A | Small molecule inhibitor | ROCK1 inhibitor |
| GSK429286A | Small molecule inhibitor | ROCK1 inhibitor |
| BMS202 | Small molecule inhibitor | PD-1 inhibitor |
| Lexatumumab (Lexa) | Monospecific Antibody | Human DR5 activator |
| AMG655 (AMG) | Monospecific Antibody | Human DR5 activator |
| Tigatuzumab (Tiga) | Monospecific Antibody | Human DR5 activator |
| KMTR2 | Monospecific Antibody | Human DR5 activator |
| MD5-1 | Monospecific Antibody | Murine DR5 activator |
| Avelumab-MD5-1 | Bispecific Antibody | Murine DR5 activator |
| Avelumab-MD5-1 | Bispecific Antibody | PD-L1 inhibitor |
| Avelumab | Bispecific Antibody | PD-L1 inhibitor |
| OKT3 | Monospecific Antibody | CD3 activator |

**Appendix Table S2.**

List of various cell lines used and generated during this study.

| Human: OVCAR-3 | Ovarian Cancer | ATCC HTB-161 |
| --- | --- | --- |
| Human: MDA-MB-436 | TNBC | ATCC HTB-130 |
| Human: MDA-MB-231 | TNBC | ATCC HTB-26 |
| Human: MDA-MB-231-2B | TNBC | ATCC HTB-26 |
| Human: PD-1 Effector Cells | Promega | J1151 |
| Human: PD-L1 aAPC/CHO-K1 Cells | Promega | J1091 |
| Human: A549 | Lung Cancer | ATCC CLL-185 |
| Human: Cavo-3 | Ovarian Cancer | ATCC HTB-75 |
| Human: HCC1806 | TNBC | ATCC CRL-2335 |
| Human: PANK1 | Pancreatic Cancer | ATCC CRL-1469 |
| Human: U87 | Brain Cancer | ATCC HTB-14 |
| Human: HCT116 | Colon Cancer | ATCC CCL247 |
| Human: Colo-205 | Colon Cancer | ATCC CCL-222 |
| Human: CHO-K cells | Stable transformed in our lab | ATCC CCL-61 |
| Mouse: 4T1 | Murine TNBC cell,  Gift from Kevin Janes, UVA, | ATCC CRL-2539 |
| Mouse: MC38 | Murine Colon Cancer,  Kind gift from Dr. Suzanne Ostrand-Rosenberg, UMBC | CVCL_B288 |
| Mouse: ID8 | Murine Ovarian Cancer, Kind gift from Melanie Rutkowski | ABC-TC3940 |
| PDX cell line : UCD52 | Dr. Chuck Harrell | VCU Pathology core |
| Mouse: MC38 Chimeric human-mouse DR-5 G4S  (Chi-G4S-DR5 cells) | Generated in our laboratory (This paper) | Human DR5 expressing murine cells |
| Mouse: 4T1 Chimeric human-mouse DR5 G4S  (Chi-G4S-DR5 cells) | Generated in our laboratory (This paper) | Human DR5 expressing murine cells |
| Mouse: 4T1 Chimeric human-mouse DR5 no G4S linker  (Chi-DR5 cells) | Generated in our laboratory (This paper) | Human DR5 expressing murine cells |
| Mouse: MC38 Chimeric human-mouse DR5 no G4S linker  (Chi-DR5 cells) | Generated in our laboratory (This paper) | Human DR5 expressing murine cells |
| Mouse: 4T1 complete human DR5 no G4S linker  (huDR5 cells) | Generated in our laboratory (This paper) | Human DR5 expressing murine cells |
| Human: MDA-MB-436 DR5Ko | Generated in our laboratory (This paper) | DR5 knockout cells |
| Human: MDA-MB-231 DR5Ko | Generated in our laboratory (This paper) | DR5 knockout cells |
| Human: MDA-MB-231-2B DR5Ko | Generated in our laboratory (This paper) | DR5 knockout cells |
| Human: MDA-MB-436 DR5 antibody Resistant | Generated in our laboratory (This paper) | DR5 resistant Cells |
| Human: MDA-MB-231 DR-5 antibody Resistant | Generated in our laboratory (This paper) | DR5 resistant Cells |
| Human: OVCAR3 DR-5 antibody Resistant | Generated in our laboratory (This paper) | DR5 resistant Cells |

Appendix Table S3.

Amino acid sequences of heavy (VH) and light (VL) chains of indicated DR5 agonists (Lexatumumab, Tigatuzumab, AMG-655, KMTR2), anti-PD-L1 (Avelumab) and other control antibodies (Farletuzumab) used in this study.

| Lexatumumab lambda (VL)  SSELTQDPAVSVALGQTVRITCQGDSLRSYYASWYQQKPGQAPVLVIYGKNNRPSGIPDRFSGSSSGNTASLTITGAQAEDEADYYCNSRDSSGNHVVFGGGTKLTVLGQPKAAPSVTLFPPSSEELQANKATLVCLISDFYPGAVTVAWKADSSPVKAGVETTTPSKQSNNKYAASSYLSLTPEQWKSHRSYSCQVTHEGSTVEKTVAPTECS  Lexatumumab IgG1 (VH)  EVQLVQSGGGVERPGGSLRLSCAASGFTFDDYGMSWVRQAPGKGLEWVSGINWNGGSTGYADSVKGRVTISRDNAKNSLYLQMNSLRAEDTAVYYCAKILGAGRGWYFDLWGKGTTVTVSSASTKGPSVFPLAPSSKSTSGGTAALGCLVKDYFPEPVTVSWNSGALTSGVHTFPAVLQSSGLYSLSSVVTVPSSSLGTQTYICNVNHKPSNTKVDKRVEPKSCDKTHTCPPCPAPEAAGGPSVFLFPPKPKDTLMISRTPEVTCVVVDVSHEDPEVKFNWYVDGVEVHNAKTKPREEQYNSTYRVVSVLTVLHQDWLNGKEYKCKVSNKALPAPIEKTISKAKGQPREPQVYTLPPSREEMTKNQVSLTCLVKGFYPSDIAVEWESNGQPENNYKTTPPVLDSDGSFFLYSKLTVDKSRWQQGNVFSCSVMHEALHNHYTQKSLSLSPGK  Tigatuzumab c-kappa (VL)  DIQMTQSPSSLSASVGDRVTITCKASQDVGTAVAWYQQKPGKAPKLLIYWASTRHTGVPSRFSGSGSGTDFTLTISSLQPEDFATYYCQQYSSYRTFGQGTKVEIKRTVAAPSVFIFPPSDEQLKSGTASVVCLLNNFYPREAKVQWKVDNALQSGNSQESVTEQDSKDSTYSLSSTLTLSKADYEKHKVYACEVTHQGLSSPVTKSFNRGEC  Tigatuzumab IgG1 (VH)  EVQLVESGGGLVQPGGSLRLSCAASGFTFSSYVMSWVRQAPGKGLEWVATISSGGSYTYYPDSVKGRFTISRDNAKNTLYLQMNSLRAEDTAVYYCARRGDSMITTDYWGQGTLVTVSSASTKGPSVFPLAPSSKSTSGGTAALGCLVKDYFPEPVTVSWNSGALTSGVHTFPAVLQSSGLYSLSSVVTVPSSSLGTQTYICNVNHKPSNTKVDKRVEPKSCDKTHTCPPCPAPEAAGGPSVFLFPPKPKDTLMISRTPEVTCVVVDVSHEDPEVKFNWYVDGVEVHNAKTKPREEQYNSTYRVVSVLTVLHQDWLNGKEYKCKVSNKALPAPIEKTISKAKGQPREPQVYTLPPSREEMTKNQVSLTCLVKGFYPSDIAVEWESNGQPENNYKTTPPVLDSDGSFFLYSKLTVDKSRWQQGNVFSCSVMHEALHNHYTQKSLSLSPGK  AMG-655 (Conatumumab) c-kappa (VL)  EIVLTQSPGTLSLSPGERATLSCRASQGISRSYLAWYQQKPGQAPSLLIYGASSRATGIPDRFSGSGSGTDFTLTISRLEPEDFAVYYCQQFGSSPWTFGQGTKVEIKRTVAAPSVFIFPPSDEQLKSGTASVVCLLNNFYPREAKVQWKVDNALQSGNSQESVTEQDSKDSTYSLSSTLTLSKADYEKHKVYACEVTHQGLSSPVTKSFNRGEC  AMG-655 (Conatumumab) IgG1 (VH)  EVQLLESGGGLVQPGGSLRLSCAASGFTFSSYIMMWVRQAPGKGLEWVSSIYPSGGITFYADTVKGRFTISRDNSKNTLYLQMNSLRAEDTAVYYCARIKLGTVTTVDYWGQGTLVTVSSASTKGPSVFPLAPSSKSTSGGTAALGCLVKDYFPEPVTVSWNSGALTSGVHTFPAVLQSSGLYSLSSVVTVPSSSLGTQTYICNVNHKPSNTKVDKKVESKYGPPCPSCPAPEFLGGPSVFLFPPKPKDTLMISRTPEVTCVVVDVSQEDPEVQFNWYVDGVEVHNAKTKPREEQFNSTYRVVSVLTVLHQDWLNGKEYKCKVSNKGLPSSIEKTISKAKGQPREPQVYTLPPSQEEMTKNQVSLTCLVKGFYPSDIAVEWESNGQPENNYKTTPPVLDSDGSFFLYSKLTVDKSRWQEGNVFSCSVMHEALHNHYTQKSLSLSPGK  KMTR2 c-kappa (VL)  EIVLTQSPATLSLSPGERATLSCRASQSVSSYLAWYQQKPGQAPRLLIYDASNRATGIPARFSGSGSGTDFTLTISSLEPEDFAVYYCQQRSNWPLTFGGGTKVEIKRTVAAPSVFIFPPSDEQLKSGTASVVCLLNNFYPREAKVQWKVDNALQSGNSQESVTEQDSKDSTYSLSSTLTLSKADYEKHKVYACEVTHQGLSSPVTKSFNRGEC  KMTR2 IgG1 (VH)  QVQLVQSGAEMKKPGASVKVSCKTSGYTFTNYKINWVRQAPGQGLEWMGWMNPDTDSTGYPQKFQGRVTMTRNTSISTAYMELSSLRSEDTAVYYCARSYGSGSYYRDYYYGMDVWGQGTTVTVSSASTKGPSVFPLAPSSKSTSGGTAALGCLVKDYFPEPVTVSWNSGALTSGVHTFPAVLQSSGLYSLSSVVTVPSSSLGTQTYICNVNHKPSNTKVDKKVEPKSCDKTHTCPPCPAPEAAGGPSVFLFPPKPKDTLMISRTPEVTCVVVDVSHEDPEVKFNWYVDGVEVHNAKTKPREEQYNSTYRVVSVLTVLHQDWLNGKEYKCKVSNKALPAPIEKTISKAKGQPREPQVYTLPPSREEMTKNQVSLTCLVKGFYPSDIAVEWESNGQPENNYKTTPPVLDSDGSFFLYSKLTVDKSRWQQGNVFSCSVMHEALHNHYTQKSLSLSPGK  Avelumab c-kappa (VL)  MGWSCIILFLVATATGVHSQSALTQPASVSGSPGQSITISCTGTSSDVGGYNYVSWYQQHPGKAPKLMIYDVSNRPSGVSNRFSGSKSGNTASLTISGLQAEDEADYYCSSYTSSSTRVFGTGTKVTVLRTVAAPSVFIFPPSDEQLKSGTASVVCLLNNFYPREAKVQWKVDNALQSGNSQESVTEQDSKDSTYSLSSTLTLSKADYEKHKVYACEVTHQGLSSPVTKSFNRGEC  Avelumab IgG4 (VH)  MGWSCIILFLVATATGVHSEVQLLESGGGLVQPGGSLRLSCAASGFTFSSYIMMWVRQAPGKGLEWVSSIYPSGGITFYADTVKGRFTISRDNSKNTLYLQMNSLRAEDTAVYYCARIKLGTVTTVDYWGQGTLVTVSSASTKGPSVFPLAPSSKSTSGGTAALGCLVKDYFPEPVTVSWNSGALTSGVHTFPAVLQSSGLYSLSSVVTVPSSSLGTQTYICNVNHKPSNTKVDKKVESKYGPPCPSCPAPEFLGGPSVFLFPPKPKDTLMISRTPEVTCVVVDVSQEDPEVQFNWYVDGVEVHNAKTKPREEQFNSTYRVVSVLTVLHQDWLNGKEYKCKVSNKGLPSSIEKTISKAKGQPREPQVYTLPPSQEEMTKNQVSLTCLVKGFYPSDIAVEWESNGQPENNYKTTPPVLDSDGSFFLYSKLTVDKSRWQEGNVFSCSVMHEALHNHYTQKSLSLSLG  Farletuzumab c-kappa (VL)  DIQLTQSPSSLSASVGDRVTITCSVSSSISSNNLHWYQQKPGKAPKPWIYGTSNLASGVPSRFSGSGSGTDYTFTISSLQPEDIATYYCQQWSSYPYMYTFGQGTKVEIKRTVAAPSVFIFPPSDEQLKSGTASVVCLLNNFYPREAKVQWKVDNALQSGNSQESVTEQDSKDSTYSLSSTLTLSKADYEKHKVYACEVTHQGLSSPVTKSFNRGEC  Farletuzumab IgG1 (VH)  EVQLVESGGGVVQPGRSLRLSCSASGFTFSGYGLSWVRQAPGKGLEWVAMISSGGSYTYYADSVKGRFAISRDNAKNTLFLQMDSLRPEDTGVYFCARHGDDPAWFAYWGQGTPVTVSSASTKGPSVFPLAPSSKSTSGGTAALGCLVKDYFPEPVTVSWNSGALTSGVHTFPAVLQSSGLYSLSSVVTVPSSSLGTQTYICNVNHKPSNTKVDKKVEPKSCDKTHTCPPCPAPEAAGGPSVFLFPPKPKDTLMISRTPEVTCVVVDVSHEDPEVKFNWYVDGVEVHNAKTKPREEQYNSTYRVVSVLTVLHQDWLNGKEYKCKVSNKALPAPIEKTISKAKGQPREPQVYTLPPSREEMTKNQVSLTCLVKGFYPSDIAVEWESNGQPENNYKTTPPVLDSDGSFFLYSKLTVDKSRWQQGNVFSCSVMHEALHNHYTQKSLSLSLPGK |
| --- |

Appendix Table S4.

Amino acid sequences of (human DR5) huDR5, Chimeric (Chi) DR5 (mouse signal peptide + human extracellular domain + mouse transmembrane + intracellular domain, see figure 5A), and Chimeric-G4S-linked (Chi-G4S) DR5 (mouse signal peptide + human extracellular domain ending with G4S linker + mouse transmembrane + intracellular domain, see figure 5A), proteins expressed in murine 4T1, ID8 and MC38 cells.

| HuDR5  MSEQRGQNAPAASGARKRHGPGPREARGARPGPRVPKTLVLVVAAVLLLVSAESALITQQDLAPQQRAAPQQKRSSPSEGLCPPGHHISEDGRDCISCKYGQDYSTHWNDLLFCLRCTRCDSGEVELSPCTTTRNTVCQCEEGTFREEDSPEMCRKCRTGCPRGMVKVGDCTPWSDIECVHKESGTKHSGEVPAVEETVTSSPGTPASPCSLSGIIIGVTVAAVVLIVAVFVCKSLLWKKVLPYLKGICSGGGGDPERVDRSSQRPGAEDNVLNEIVSILQPTQVPEQEMEVQEPAEPTGVNMLSPGESEHLLEPAEAERSQRRRLLVPANEGDPTETLRQCFDDFADLVPFDSWEPLMRKLGLMDNEIKVAKAEAAGHRDTLYTMLIKWVNKTGRDASVHTLLDALETLGERLAKQKIEDHLLSSGKFMYLEGNADSAMS  Chi-DR5  MSEQRGQNAPAASGARKRHGPGPREARGARPGPRVPKTLVLVVAAVLLLVSAESALITQQDLAPQQRAAPQQKRSSPSEGLCPPGHHISEDGRDCISCKYGQDYSTHWNDLLFCLRCTRCDSGEVELSPCTTTRNTVCQCEEGTFREEDSPEMCRKCRTGCPRGMVKVGDCTPWSDIECVHKESGTKHSGEVPAVEETVTSSPGTPASPCSLSGLWIGLLVPVVLLIGALLVWKTGAWRQWLLCIKRGCERDPESANSVHLSLLDRQTSSTTNDSNHNTEPGKTQKTGKKLLVPVNGNDSADDLKFIFEYCSDIVPFDSWNRLMRQLGLTDNQIQMVKAETLVTREALYQMLLKWRHQTGRSASINHLLDALEAVEERDAMEKIEDYAVKSGRFTYQNAAAQPETGPGGSQCV  Chi-G4S DR5  MEPPGPSTPTASAAARADHYTPGLRPLPKRRLLYSFALLLAVLQAVFVPVTAITQQDLAPQQRAAPQQKRSSPSEGLCPPGHHISEDGRDCISCKYGQDYSTHWNDLLFCLRCTRCDSGEVELSPCTTTRNTVCQCEEGTFREEDSPEMCRKCRTGCPRGMVKVGDCTPWSDIECVHKESGTKHSGEVPAVEETVTSSPGTPASPCSGGGGSLGLWIGLLVPVVLLIGALLVWKTGAWRQWLLCIKRGCERDPESANSVHLSLLDRQTSSTTNDSNHNTEPGKTQKTGKKLLVPVNGNDSADDLKFIFEYCSDIVPFDSWNRLMRQLGLTDNQIQMVKAETLVTREALYQMLLKWRHQTGRSASINHLLDALEAVEERDAMEKIEDYAVKSGRFTYQNAAAQPETGPGGSQCV |
| --- |

**4) References for Material and Methods**

Asiedu MK, Ingle JN, Behrens MD, Radisky DC, Knutson KL (2011) TGFbeta/TNF(alpha)-mediated epithelial-mesenchymal transition generates breast cancer stem cells with a claudin-low phenotype. *Cancer Res* 71: 4707-4719

Calve S, Witten AJ, Ocken AR, Kinzer-Ursem TL (2016) Incorporation of non-canonical amino acids into the developing murine proteome. *Sci Rep* 6: 32377

Durocher Y, Butler M (2009) Expression systems for therapeutic glycoprotein production. *Curr Opin Biotechnol* 20: 700-707

Graves JD, Kordich JJ, Huang TH, Piasecki J, Bush TL, Sullivan T, Foltz IN, Chang W, Douangpanya H, Dang T *et al* (2014) Apo2L/TRAIL and the death receptor 5 agonist antibody AMG 655 cooperate to promote receptor clustering and antitumor activity. *Cancer Cell* 26: 177-189

Leelatian N, Doxie DB, Greenplate AR, Sinnaeve J, Ihrie RA, Irish JM (2017) Preparing Viable Single Cells from Human Tissue and Tumors for Cytomic Analysis. *Curr Protoc Mol Biol* 118: 25C 21 21-25C 21 23

Li F, Ravetch JV (2012) Apoptotic and antitumor activity of death receptor antibodies require inhibitory Fcgamma receptor engagement. *Proc Natl Acad Sci U S A* 109: 10966-10971

Shen B, Fang Y, Wu N, Gould SJ (2011) Biogenesis of the posterior pole is mediated by the exosome/microvesicle protein-sorting pathway. *J Biol Chem* 286: 44162-44176

Shivange G, Urbanek K, Przanowski P, Perry JSA, Jones J, Haggart R, Kostka C, Patki T, Stelow E, Petrova Y *et al* (2018) A Single-Agent Dual-Specificity Targeting of FOLR1 and DR5 as an Effective Strategy for Ovarian Cancer. *Cancer Cell* 34: 331-345 e311

Takeda K, Kojima Y, Ikejima K, Harada K, Yamashina S, Okumura K, Aoyama T, Frese S, Ikeda H, Haynes NM *et al* (2008) Death receptor 5 mediated-apoptosis contributes to cholestatic liver disease. *Proc Natl Acad Sci U S A* 105: 10895-10900

Tan YS, Lei YL (2019) Isolation of Tumor-Infiltrating Lymphocytes by Ficoll-Paque Density Gradient Centrifugation. *Methods Mol Biol* 1960: 93-99

Wang L, Yu C, Yang Y, Gao K, Wang J (2017) Development of a robust reporter gene assay to measure the bioactivity of anti-PD-1/anti-PD-L1 therapeutic antibodies. *J Pharm Biomed Anal* 145: 447-453

Whitford P, Mallon EA, George WD, Campbell AM (1990) Flow cytometric analysis of tumour infiltrating lymphocytes in breast cancer. *Br J Cancer* 62: 971-975

Wilson NS, Yang B, Yang A, Loeser S, Marsters S, Lawrence D, Li Y, Pitti R, Totpal K, Yee S *et al* (2011) An Fcgamma receptor-dependent mechanism drives antibody-mediated target-receptor signaling in cancer cells. *Cancer Cell* 19: 101-113

Wollebo HS, Woldemichaele B, White MK (2013) Lentiviral transduction of neuronal cells. *Methods Mol Biol* 1078: 141-146

Wu JJ, Zhang XD, Gillespie S, Hersey P (2005) Selection for TRAIL resistance results in melanoma cells with high proliferative potential. *FEBS Lett* 579: 1940-1944

Zhang F, Qi X, Wang X, Wei D, Wu J, Feng L, Cai H, Wang Y, Zeng N, Xu T *et al* (2017) Structural basis of the therapeutic anti-PD-L1 antibody atezolizumab. *Oncotarget* 8: 90215-90224
