# Supplementary material for: Unexpected PD‐L1 immune evasion mechanism in TNBC, ovarian, and other solid tumors by DR5 agonist antibodies
Source: EMBO Mol Med. 2021 Feb 15;13(3):e12716. doi: 10.15252/emmm.202012716 (PMC7933954; doi:10.15252/emmm.202012716)
Supplement: Supplementary file 2 — Expanded View Figures PDF [file EMMM-13-e12716-s002.pdf]

# Expanded View Figures

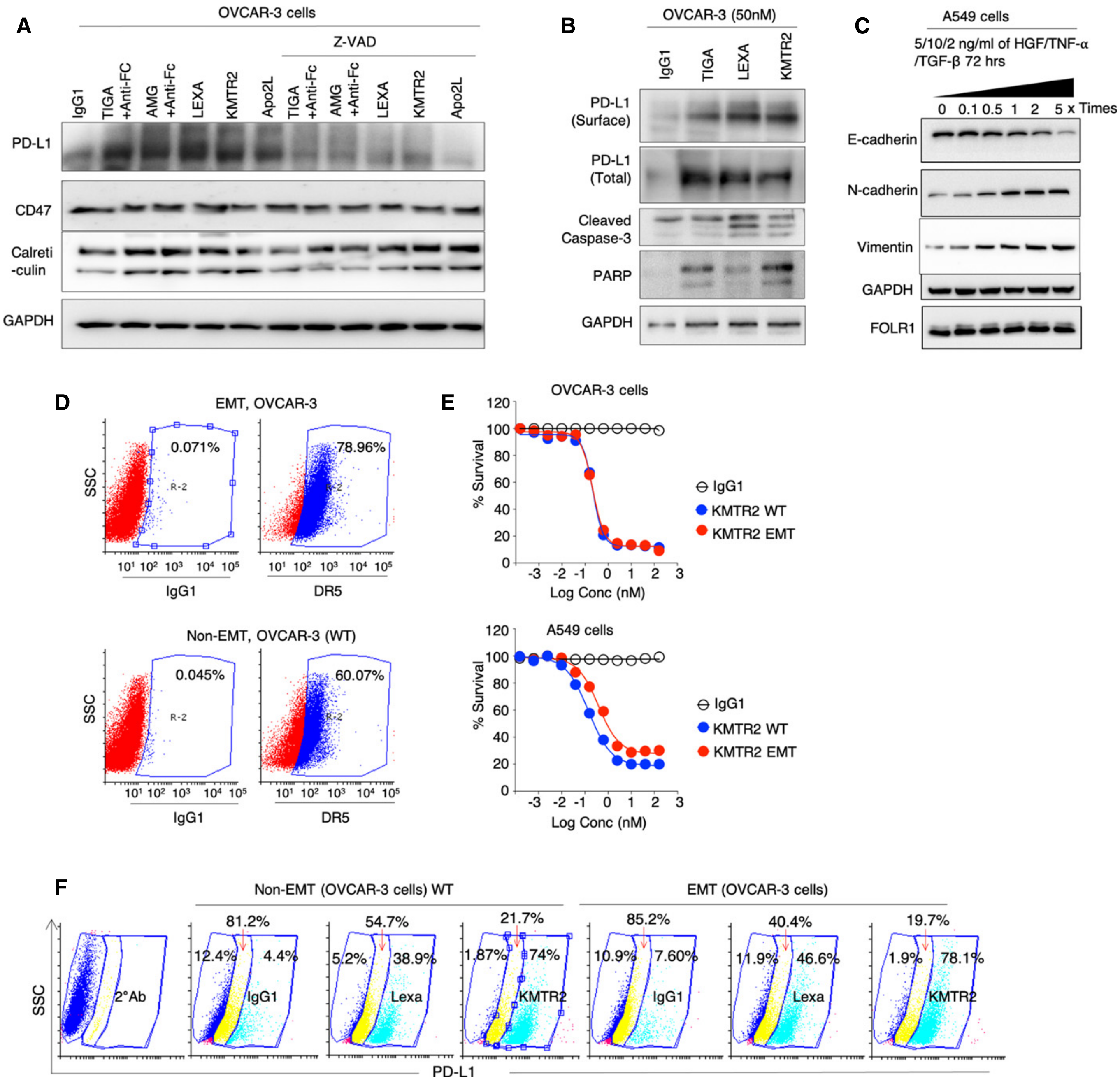


**Figure EV1. DR5 agonist induced PD-L1 stabilization during transient epithelial-to-mesenchymal transitions.**

A Immunoblotting analysis of PD-L1, CD47, and calreticulin from OVCAR-3 cell lysates treated with indicated DR5 agonist antibodies ± caspase inhibitor Z-VAD. GAPDH is loading control.

B Immunoblotting of PD-L1, caspase-3, and PARP following surface biotinylation of PD-L1 from OVCAR-3 cells after indicated DR5 agonist treatments.

C Immunoblotting of N-cadherin, E-cadherin, FOLR1, and vimentin from A549 cells after treatment with indicated growth factor (HGF, TNF-α, and TGF-β) for indicated times to induce transient epithelial-to-mesenchymal transitions (EMTs).

D Similar to (C) except OVCAR3 cells were used and were analyzed for surface DR5 expression prior and after transient EMT induction.

E Cell viability assays of OVCAR3 and A549 cells in EMT and non-EMT conditions after treatment with KMTR2.

F PD-L1 flow cytometry analysis of OVCAR3 cells in non-EMT and EMT conditions after treatment with indicated DR5 agonist antibodies.

© 2021 The Authors

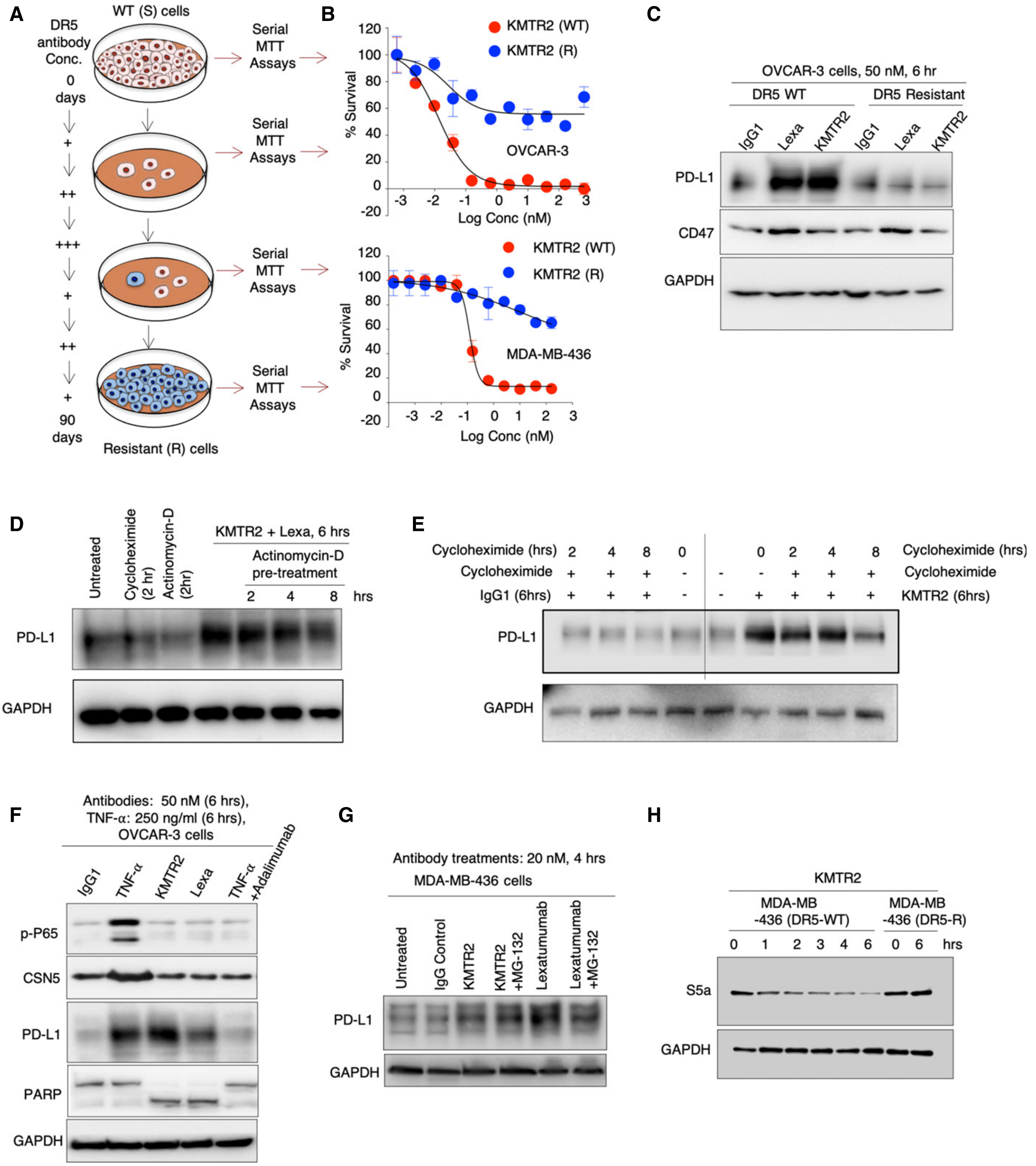


**Figure EV2.**

 © 2021 The Authors

◀ **Figure EV2. DR5-resistant tumors cells, transcription and translation inhibition do not stabilize PD-L1 upon DR5 agonist antibody treatments.**

A Schematic of DR5 agonist resistant cell line generation. DR5 expressing WT cells were treated with varying concentrations of lexatumumab to select the resistant colonies. Selected colonies were continuously treated with lexatumumab to generate resistant clones.
B Cell survival assays confirming generation of DR5-resistant cell lines ($n$ = 2), Error bar indicates SD.
C Total PD-L1 and CD47 blotting analysis from DR5-resistant OVCAR-3 cell lines after indicated DR5 agonist treatment (50 nM, 6 h).
D MDA-MB-436 cells were pre-treated for 0, 2, 4, and 8 h with actinomycin-D followed by KMTR2 + lexa for 6 h. Left 3 lanes are controls. Lysates were analyzed for PD-L1 levels.
E MDA-MB-436 cells were pre-treated for 0, 2, 4, and 8 h with cycloheximide followed by KMTR2 for 6 h. After 6 h of KMTR2 treatment, lysates were analyzed for PD-L1 levels.
F OVCAR-3 cells were treated with TNFα and indicated DR5 agonists for indicated times. Lysates were analyzed for PD-L1, CSN5, phosphorylated p65 and PARP. GAPDH is loading control.
G MDA-MB-436 cells were treated with indicated DR5 agonist ± MG132. Lysates were analyzed for total PD-L1. GAPDH is loading control.
H WT DR5-sensitive (DR5-S) and DR5-resistant (DR5-R) MDA-MB-436 cells were treated for indicated times with KMTR2. Total lysates were analyzed for S5a/PSMD4, a subunit of 26S proteasome regulatory complex.

© 2021 The Authors 

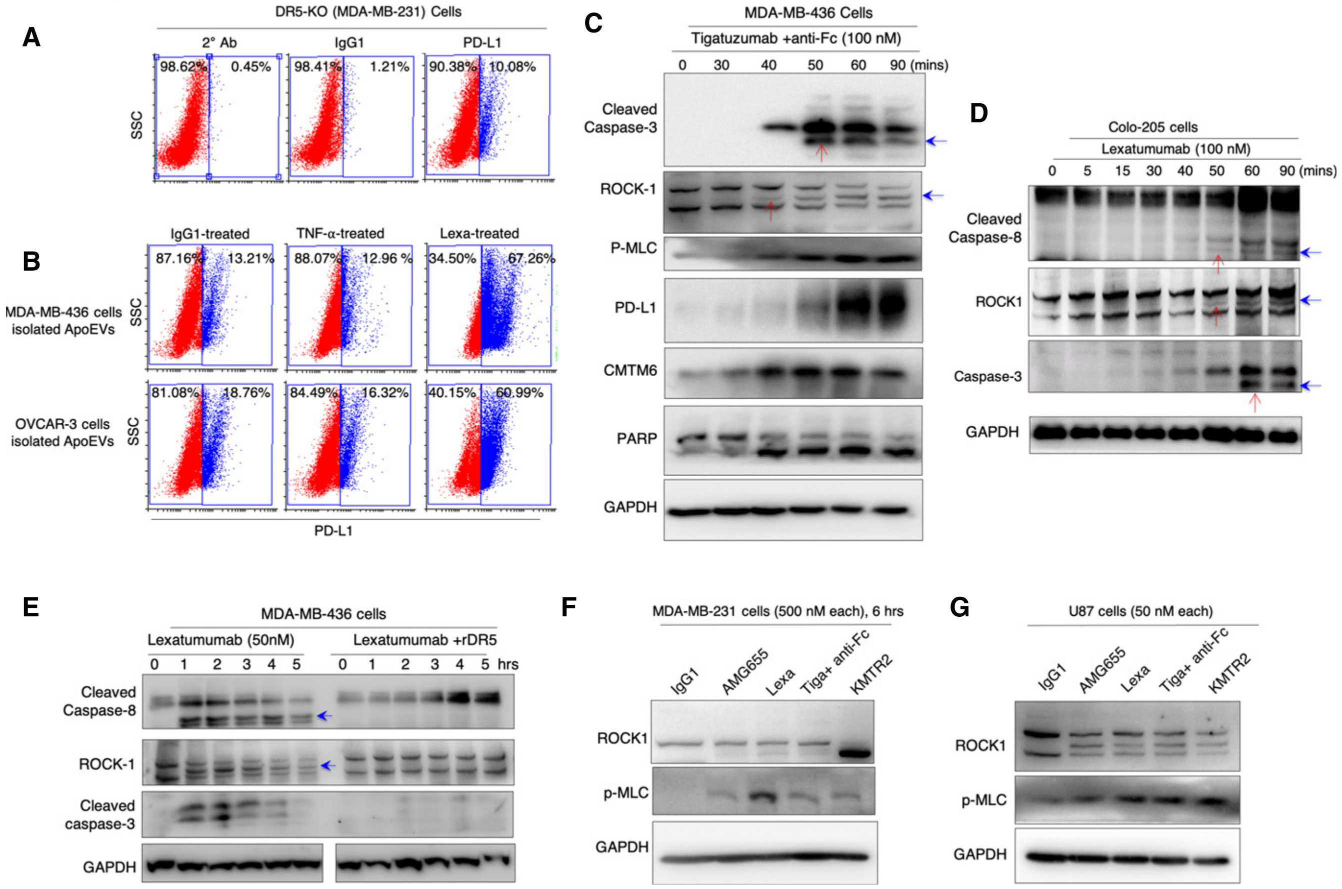


**Figure EV3. TNF-α stabilized PD-L1 is not shuttled to ApoEVs and DR5 agonist activates ROCK1.**

A, B ApoEVs were isolated from DR5-WT MDA-MB-436 and OVCAR-3 cells after treatment with IgG1, TNF-α and lexatumumab. Isolated ApoEVs were incubated on to DR5-KO (MDA-MB-231) cells. After 24 h surface PD-L1 of DR5-KO cells was analyze by flow cytometry. Top panel (A) shows secondary antibody control, IgG1 treatment control and basal level PD-L1 levels on MDA-MB-231 DR5 KO cells. Bottom panel (B) shows surface PD-L1 after ApoEV treatment.

C Tigatuzumab (anti-DR5) + anti-Fc treated MDA-MB-436 cell lysates for indicated early time points were analyzed for caspase-3, ROCK1, pMLC, PARP, PD-L1, and CMTM6 as indicated. Red arrows indicate sequential kinetics of caspase-3 and ROCK1 activation. Blue arrows indicate cleaved ROCK1 and caspase-3.

D Lexatumumab-treated Colo-205 cell lysates for indicated early time points were analyzed for cleaved caspase-8, ROCK1, and cleaved caspase-3. Red arrows indicate sequential kinetics of caspase-8, ROCK1, and caspase-3 activation. Blue arrows indicate cleaved caspase-8, ROCK1, and caspase-3.

E Lexatumumab ± rDR5-treated MDA-MB-436 cell lysates for indicated time points were analyzed for cleaved caspase-8, ROCK1, and cleaved caspase-3. Blue arrows indicate cleaved caspase-8 and ROCK1.

F, G Various clinical DR5 agonist antibody-mediated cleavage and activation of ROCK1 in MDA-MB-231 and U87 cells as confirmed by increased phosphorylation of myosin light chain, a ROCK1 substrate.

 © 2021 The Authors

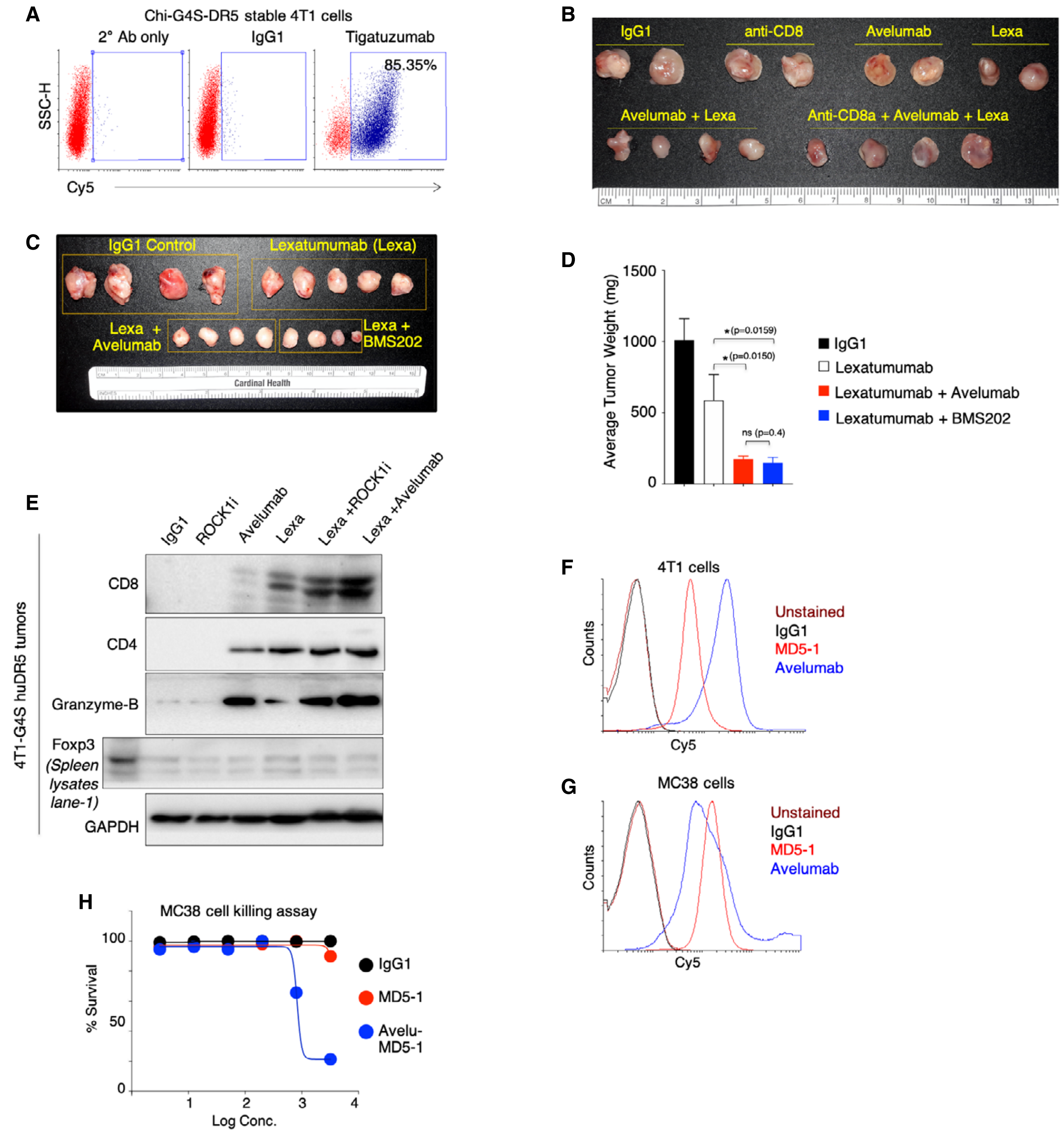
A
Chi-G4S-DR5 stable 4T1 cells
2° Ab only
IgG1
Tigatuzumab
85.35%
SSC-H
Cy5
B
IgG1
anti-CD8
Avelumab
Lexa
Avelumab + Lexa
Anti-CD8a + Avelumab + Lexa
C
IgG1 Control
Lexatumumab (Lexa)
Lexa + Avelumab
Lexa + BMS202
Cardinal Health
D
Average Tumor Weight (mg)
*(p=0.0159)
*(p=0.0150)
ns (p=0.4)
IgG1
Lexatumumab
Lexatumumab + Avelumab
Lexatumumab + BMS202
E
IgG1
ROCK1i
Avelumab
Lexa
Lexa +ROCK1i
Lexa +Avelumab
4T1-G4S huDR5 tumors
CD8
CD4
Granzyme-B
Foxp3 (Spleen lysates lane-1)
GAPDH
F
4T1 cells
Counts
Cy5
Unstained
IgG1
MD5-1
Avelumab
G
MC38 cells
Counts
Cy5
Unstained
IgG1
MD5-1
Avelumab
H
MC38 cell killing assay
% Survival
Log Conc.
IgG1
MD5-1
Avelu-MD5-1

**Figure EV4.**

**Figure EV4. Both PD-1 and PD-L1 blockade improve anti-tumor function and confirmation of activated immune infiltration DR5 + ROCK1i and DR5 + PD-L1 co-targeting.**

A Chi-G4S-DR5 stable 4T1 cell were analyzed for DR5 expression using clinical tigatuzumab antibody in flow cytometry assay.
B Chi-G4S-DR5 stable 4T1 tumors were treated with either single antibodies (IgG1, CD8 cells depleting anti-CD8a, avelumab, lexatumumab) or in combinations (avelumab + lexatumumab or avelumab + lexatumumab+ anti-CD8a). 6, 100 μg dose of each antibodies was injected, and tumors were harvested and imaged at same time (See Fig 5M for tumor weight quantitation).
C Chi-G4S-DR5 stable 4T1 tumors were treated with IgG1, lexatumumab, and lexatumumab in combination of either avelumab (anti-PD-L1) or BMS202, a PD-1 inhibitor. After six doses, tumors were harvested and imaged at same time.
D Quantitation of average tumor weight as shown in (C).
E Chi-G4S-DR5 stable 4T1 tumors harboring mice were treated (6 total doses) lexatumumab, avelumab, ROCK1i, lexatumumab + ROCKi, and avelumab + lexatumumab and IgG1 control as indicated. Harvested tumors homogenized followed by quantitation. Protein lysates were run on SDS–PAGE followed by immunoblotting using indicated CD8, CD4, Foxp3, and granzyme-b antibody. GAPDH is loading control.
F FACS histogram showing binding of anti-mouse DR5 antibody (MD5-1) and anti-mouse cross-reactive clinical PD-L1 antibody (avelumab) to 4T1 cells.
G FACS histogram showing binding of anti-mouse DR5 antibody (MD5-1) and anti-mouse cross-reactive clinical PD-L1 antibody (avelumab) to MC38 cells.
H Cell killing assay of MC38 cells treated with murine DR5 agonist MD5-1 and bispecific avelu-MD5 antibody.

Data information: Mean ± SD. Statistical significance in (D) was determined using two-tailed Mann–Whitney test (*$P < 0.05$).

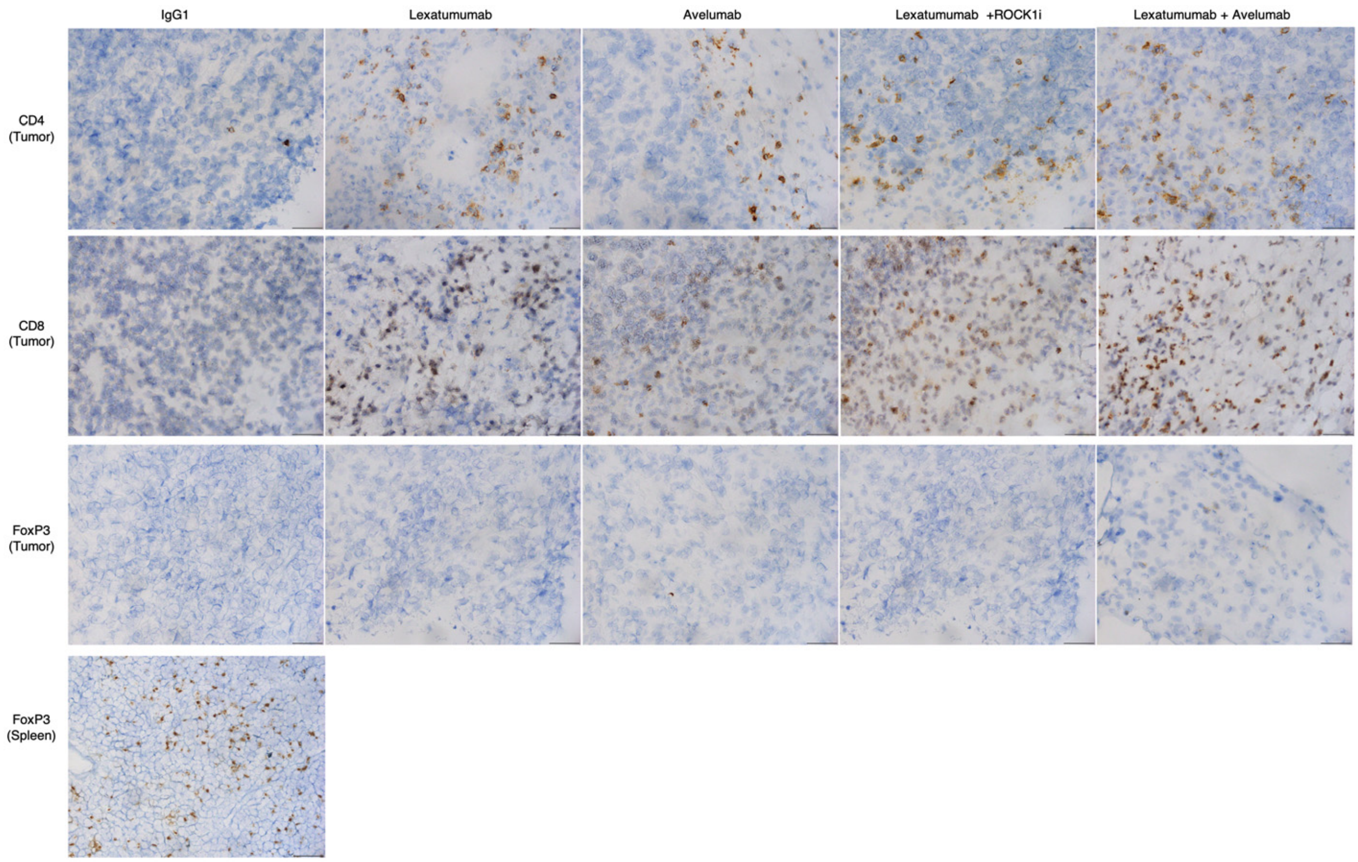


**Figure EV5. CD8$^+$ and CD4$^+$ T-cell tumor infiltration is enhanced upon DR5 agonist treatments.**

Chi-G4S-DR5 stable MC38 tumors harboring mice were treated (6 total doses) lexatumumab, avelumab, lexatumumab + ROCKi, and avelumab + lexatumumab and IgG1 control as indicated. Mouse tumors were collected at 100–200 $mm^3$ & embedded in O.C.T. to make blocks. Tumor sections were stained with antibodies (CD8, CD4, FoxP3) and counter-stained with hematoxylin as described in Methods section. One representative tumor from Fig 6A ($n$ = 3) was processed for IHC studies after indicated treatments. Multiple images were taken from IHC samples, and one representative image is shown for each treatment. Since tumors were almost negative against FoxP3, a marker of regulatory T cells (see Figs 6D and EV4E), animal spleen tissue was processed for IHC analysis as a positive control. Images were acquired at 50 × magnification for each tumor sample, and scale bar at the bottom of image is 20 μm.


© 2021 The Authors
