# Supplementary figures and images for: Unexpected PD‐L1 immune evasion mechanism in TNBC, ovarian, and other solid tumors by DR5 agonist antibodies
Source: EMBO Mol Med. 2021 Feb 15;13(3):e12716. doi: 10.15252/emmm.202012716 (PMC7933954; doi:10.15252/emmm.202012716)

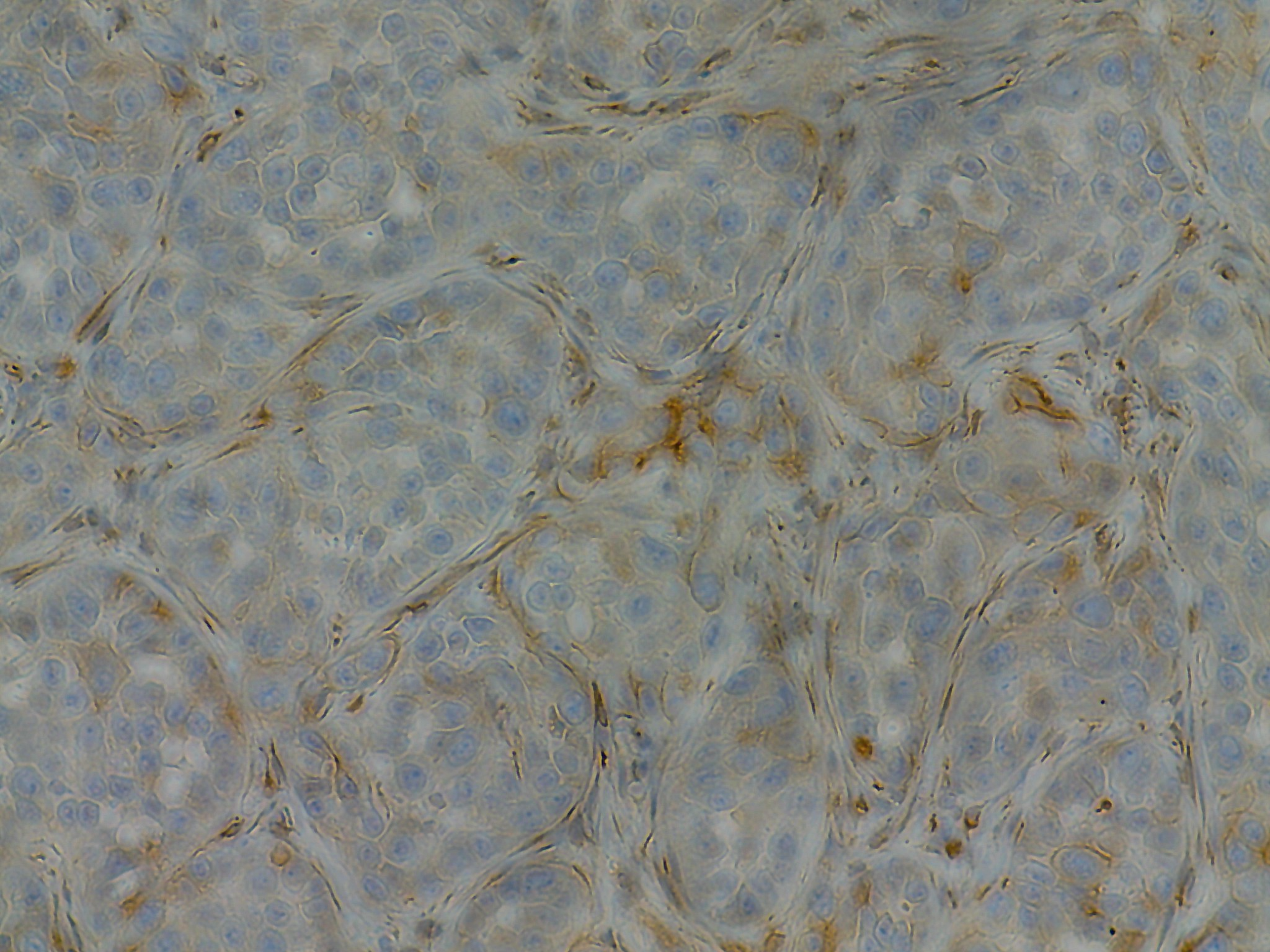

Supplement: Supplementary file 5 — Source Data for Figure 1 [file EMMM-13-e12716-s003.zip › Figure 1L (1).tiff]

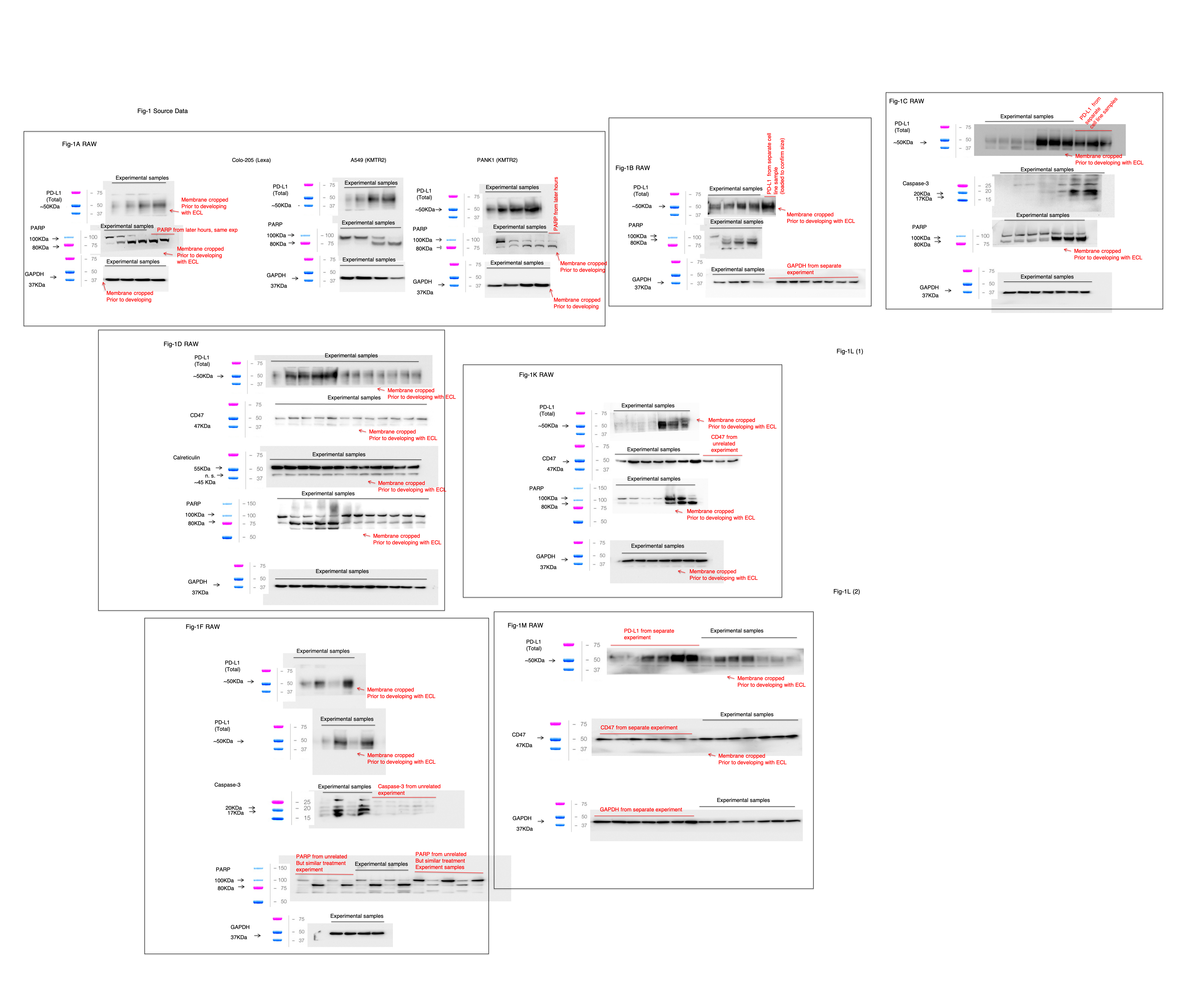

Supplement: Supplementary file 5 — Source Data for Figure 1 [file EMMM-13-e12716-s003.zip › Figure 1_Source Data.png]

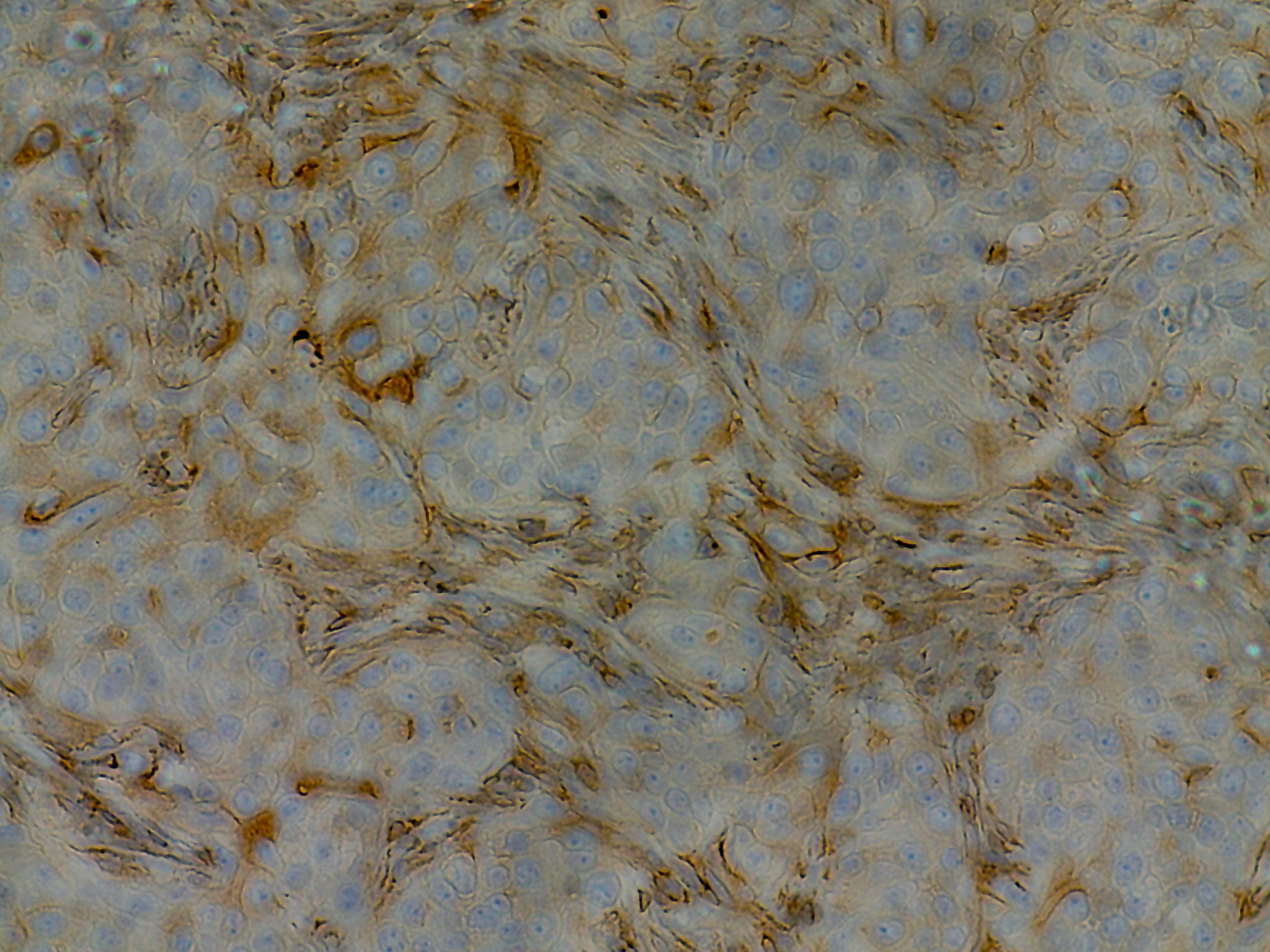

Supplement: Supplementary file 5 — Source Data for Figure 1 [file EMMM-13-e12716-s003.zip › Figure 1L (2).tiff]

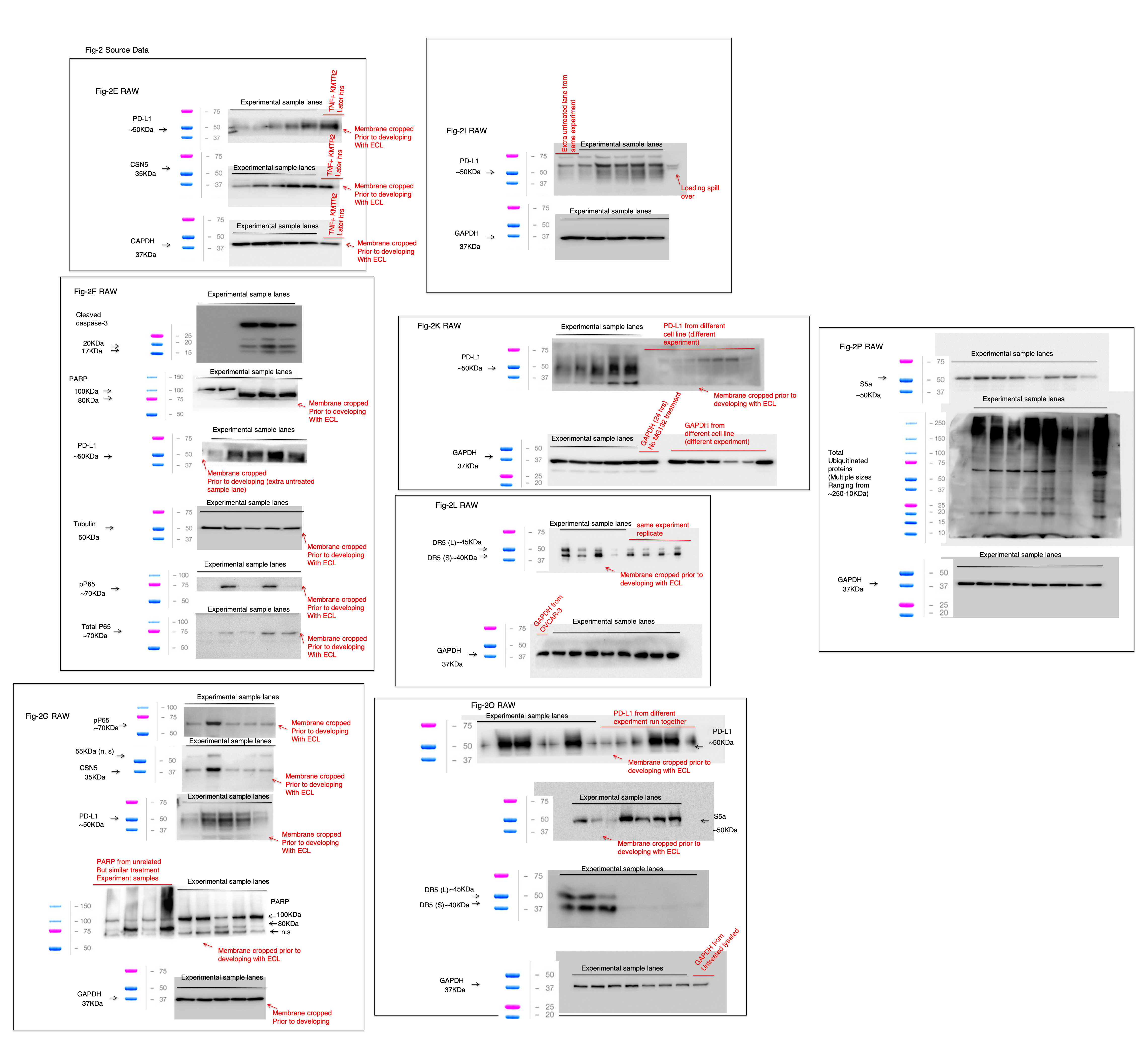

Supplement: Supplementary file 6 — Source Data for Figure 2 [file EMMM-13-e12716-s004.zip › Figure 2_Source Data.png]

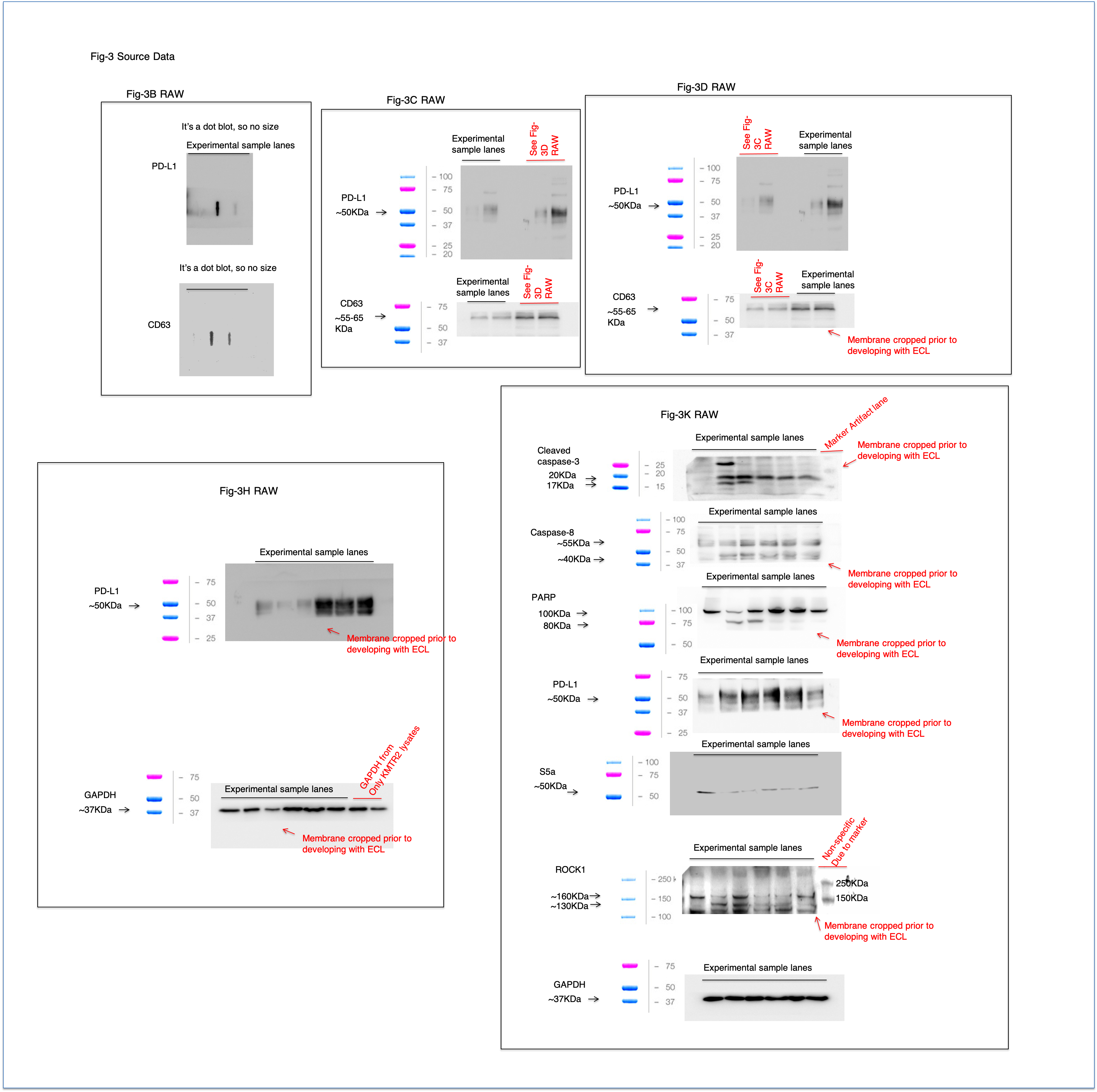

Supplement: Supplementary file 7 — Source Data for Figure 3 [file EMMM-13-e12716-s005.zip › Figure 3_Source Data.png]

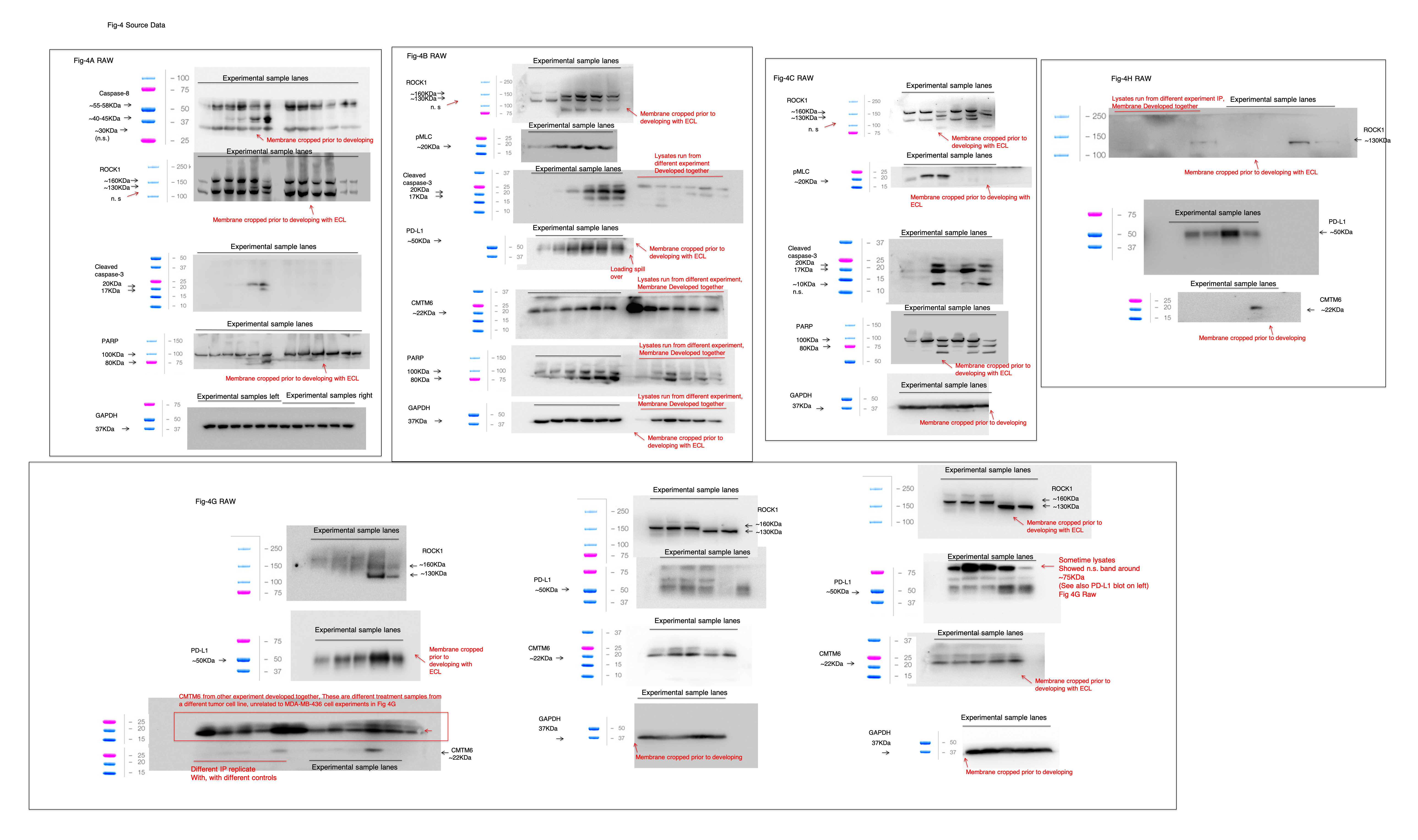

Supplement: Supplementary file 8 — Source Data for Figure 4 [file EMMM-13-e12716-s006.zip › Figure 4_Source Data.png]

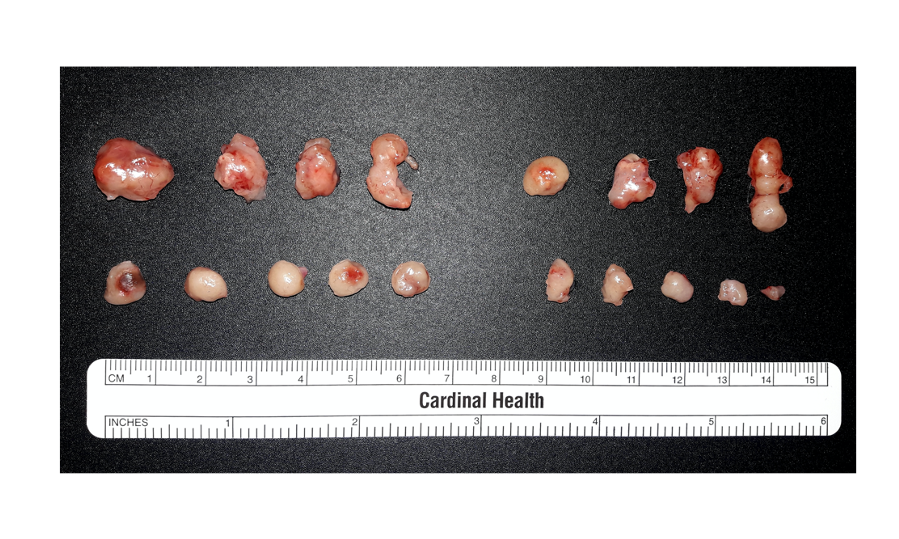

Supplement: Supplementary file 9 — Source Data for Figure 5 [file EMMM-13-e12716-s007.zip › Figure 5 Source Data/Fig 5H.tiff]

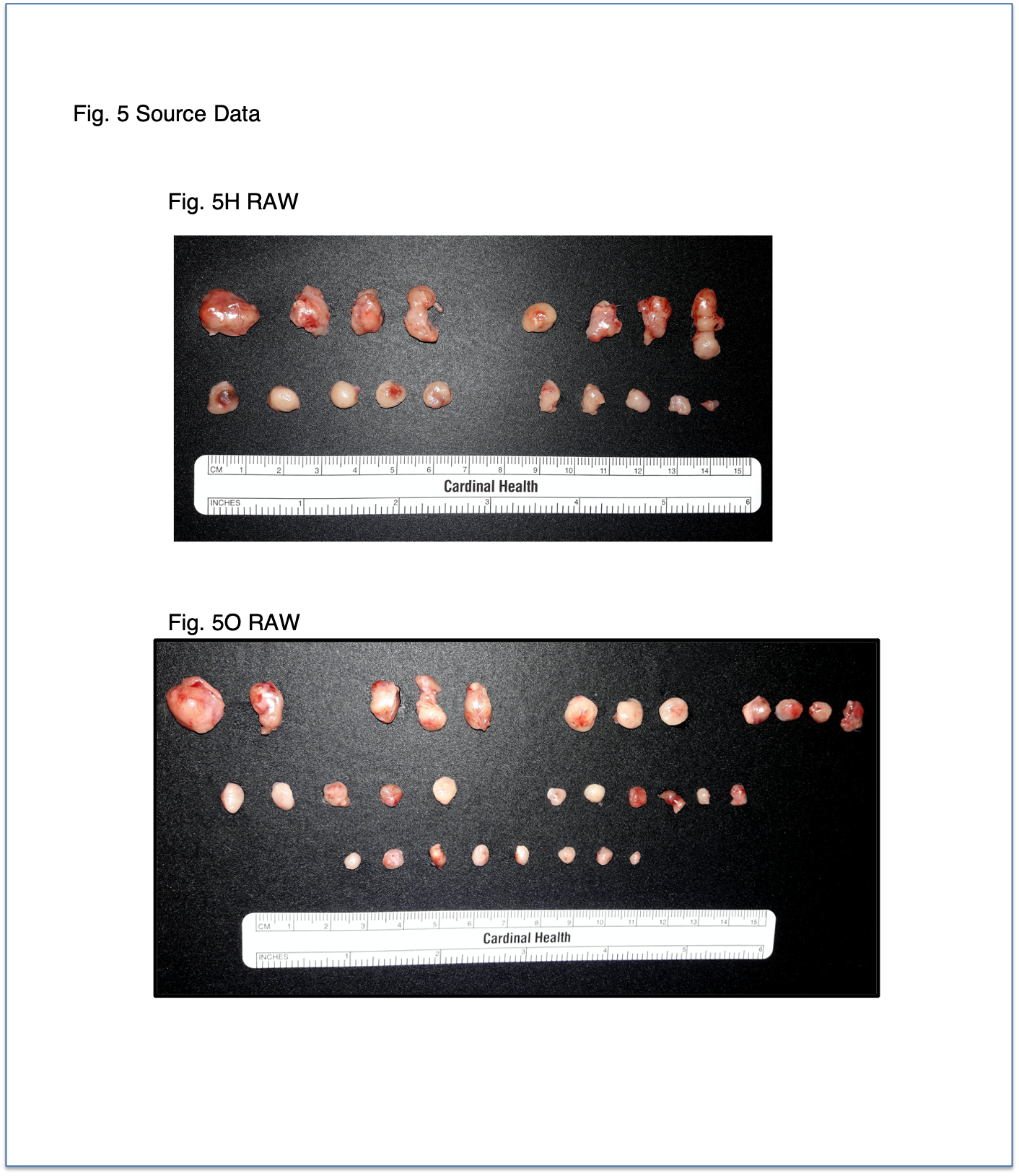

Supplement: Supplementary file 9 — Source Data for Figure 5 [file EMMM-13-e12716-s007.zip › Figure 5 Source Data/SD_Figure 5.tiff]

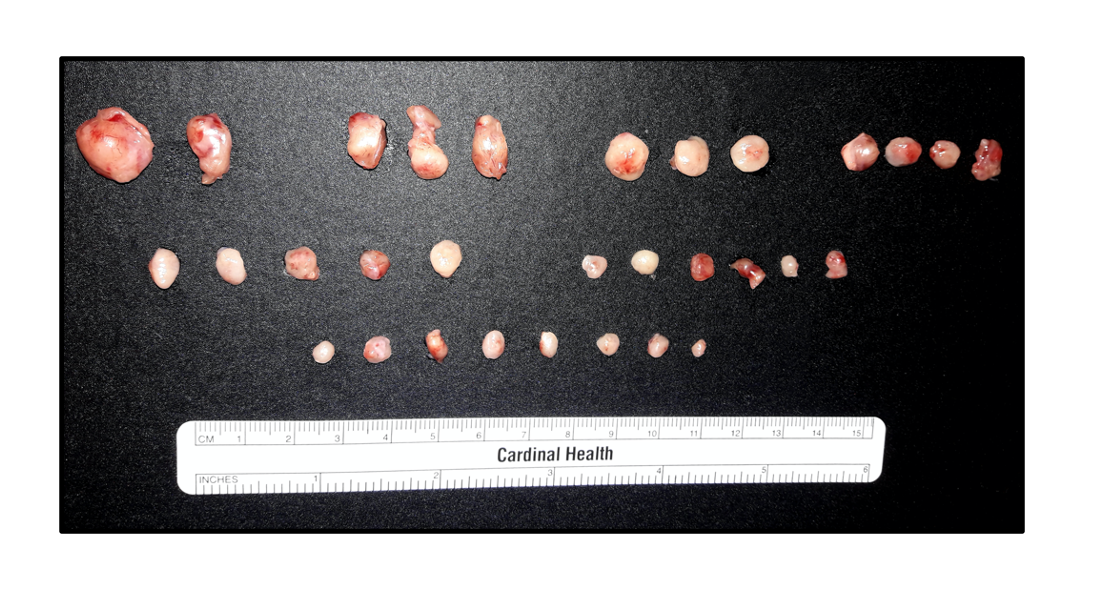

Supplement: Supplementary file 9 — Source Data for Figure 5 [file EMMM-13-e12716-s007.zip › Figure 5 Source Data/Fig 5O.tiff]

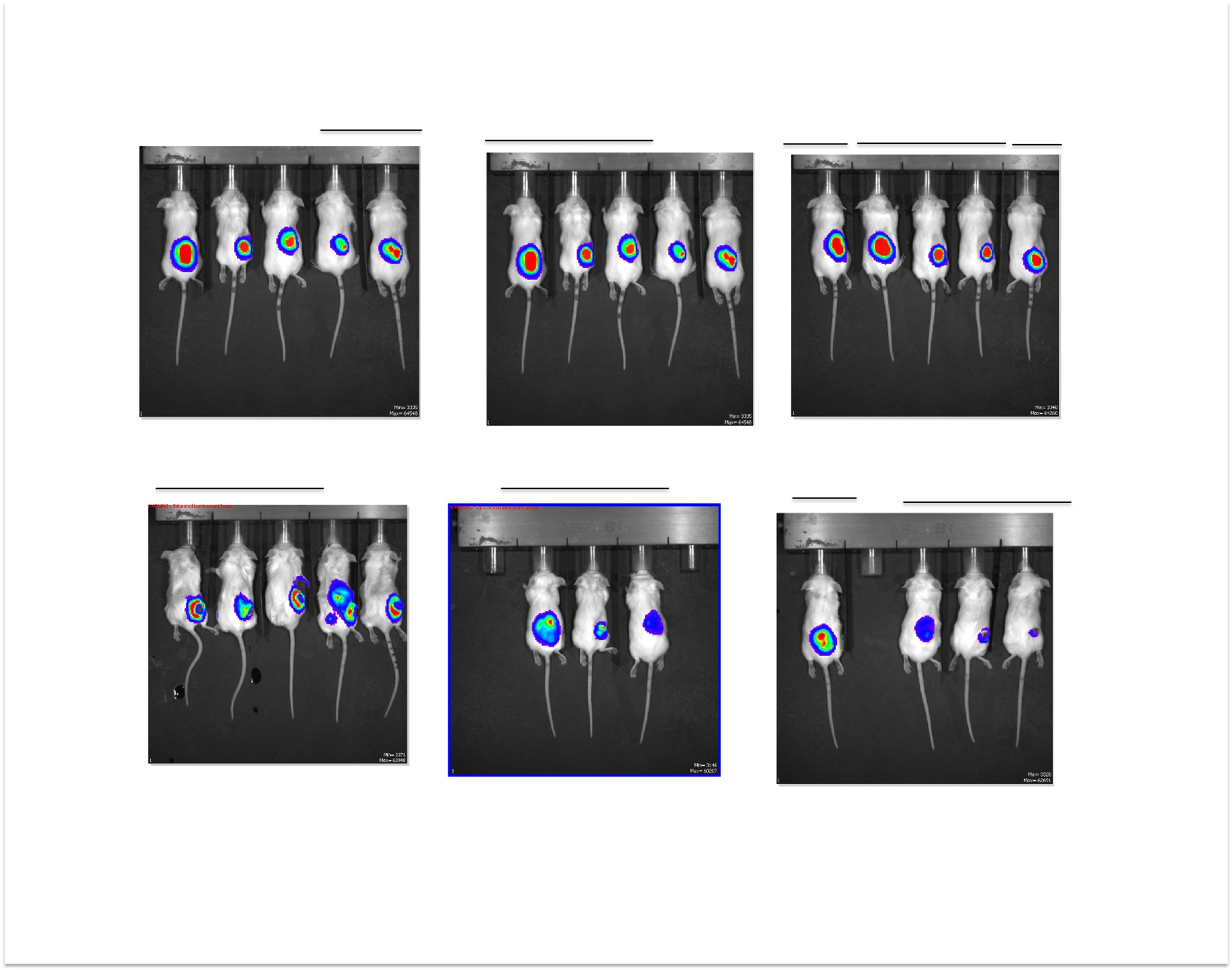

Supplement: Supplementary file 10 — Source Data for Figure 6 [file EMMM-13-e12716-s008.zip › Figure 6 Source Data/Fig 6I.tiff]

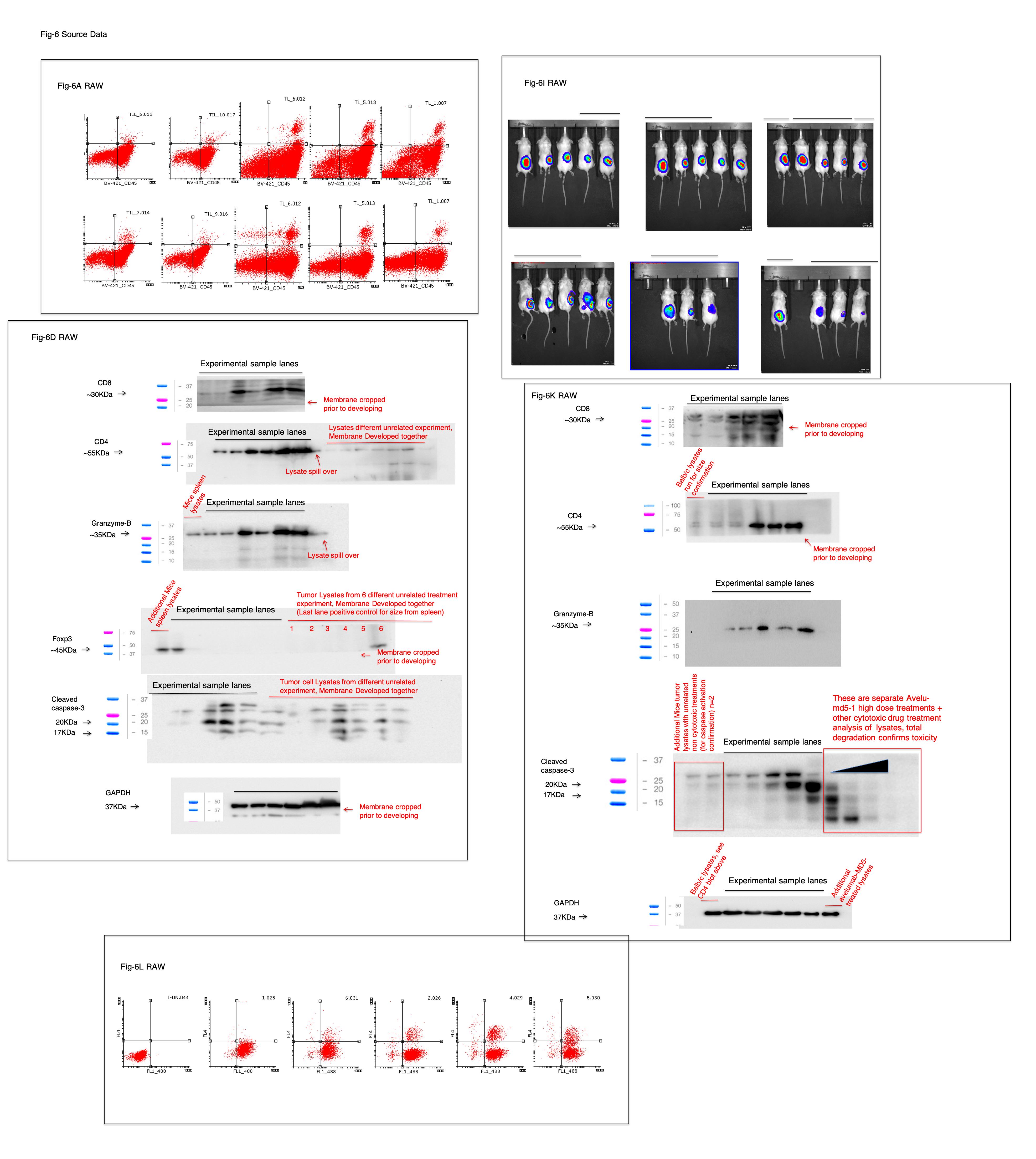

Supplement: Supplementary file 10 — Source Data for Figure 6 [file EMMM-13-e12716-s008.zip › Figure 6 Source Data/Figure 6_Source Data.png]
